# The stability of memristive multidirectional associative memory neural networks with time-varying delays in the leakage terms via sampled-data control

**DOI:** 10.1371/journal.pone.0204002

**Published:** 2018-09-24

**Authors:** Weiping Wang, Xin Yu, Xiong Luo, Long Wang, Lixiang Li, Jürgen Kurths, Wenbing Zhao, Jiuhong Xiao

**Affiliations:** 1 School of Computer and Communication Engineering, University of Science and Technology Beijing, Beijing, China; 2 Beijing Key Laboratory of Knowledge Engineering for Materials Science, Beijing, China; 3 Institute of Physics, Humboldt-University, Berlin, Germany; 4 Information Security Center, State Key Laboratory of Networking and Switching Technology, Beijing University of Posts and Telecommunications, Beijing, China; 5 Potsdam Institute for Climate Impact Research, Potsdam, Germany; 6 Department of Electrical Engineering and Computer Science, Cleveland State University, Cleveland, Ohio, United States of America; 7 School of Automation and Electrical Engineering, University of Science and Technology Beijing, Beijing, China; Shandong University of Science and Technology, CHINA

## Abstract

In this paper, we propose a new model of memristive multidirectional associative memory neural networks, which concludes the time-varying delays in leakage terms via sampled-data control. We use the input delay method to turn the sampling system into a continuous time-delaying system. Then we analyze the exponential stability and asymptotic stability of the equilibrium points for this model. By constructing a suitable Lyapunov function, using the Lyapunov stability theorem and some inequality techniques, some sufficient criteria for ensuring the stability of equilibrium points are obtained. Finally, numerical examples are given to demonstrate the effectiveness of our results.

## Introduction

Associative memory is one of the most important activities of human brains. It includes one-to-many association, many-to-one association and many-to-many association. Due to the complexity of human brains, many-to-many associative memory is more suitable for simulating the associative memory process of human brains than one-to-many association or many-to-one association.

Multidirectional associative memory neural networks(MAMNNs) were proposed by Japanese scholars in 1990 [1]. They are used to realize many-to-many association. Moreover, MAMNNs are the extension of bidirectional associative memory neural networks(BAMNNs), and they are similar in structure, i.e. there is no connection between the neurons in the same field, but there exist interconnections between the neurons from different fields. In recent years, some studies have analyzed and dealt with MAMNNs in [2–4]. In [2], the authors proposed a multi-valued exponential associative memory model, and they analyzed the stability of this system. The global exponential stability of MAMNNs with time-varying delays were analyzed in [3]. In addition, MAMNNs with almost periodic coefficients and continuously distributed delays were studied in [4]. So far, there have been few results on the stability of MAMNNs, therefore, it is significant to analyze the stability of MAMNNs.

Due to the characteristics of a memristor, it has been found to be the best device for simulating variable synaptic weights of human brains. Therefore, according to using the memristors in neural networks(NNs) instead of resistors, memristive neural networks(MNNs) was designed in [5, 6]. Since then, the dynamic behaviors of MNNs have attracted the attention of many researchers in [7–10], and they have been widely applied to associative memory [11], medical image processing [12], etc. Meanwhile, BAMNNs as a special case of MAMNNs, memristive bidirectional associative memory neural networks(MBAMNNs) have been extensively studied in [13–17]. As an extension of MBAMNNs, the study of memristive multidirectional associative memory neural networks(MMAMNNs) have attracted the attention of researchers [18]. However, it is worth noting that, because of the complexity of MMAMNNs, their research results are few. Thus, it is meaningful to analyze the dynamic behaviors of MMAMNNs.

It is well known that stability of systems plays an important role due to their potential applications to image encryption [19, 20], associative memory [11], medical image processing [12], information storage [18], etc. In the past few years, the stability of MNNs and MBAMNNs have attracted the attention of many researchers [21–24]. Global exponential stability of MNNs with impulse time window and time-varying delays was discussed in [21]. The problem of exponential stability for switched MNNs with time-varying delays was studied in [22]. The theoretical results on the global asymptotic stability and synchronization of a class of fractional-order MNNs with multiple delays were analyzed in [23]. Based on above discussions, the existence, uniqueness and exponential stability for complex-valued MBAMNNs with time delays were studied in [24]. As we all know, a stable equilibrium or a periodic solution is stored as an associative memory pattern. The storage capacity of a system is the collection of associative memory patterns. In other words, the more equilibrium points, the larger the storage capacity. Recently, some results about the multistability of MNNs have been found in [25, 26]. At present, there are few literatures about the stability of MMAMNNs, accordingly, stability and multistability of MMAMNNs are still a problem that deserves investigation.

Delays play an important role in the system. The time-varying delays are inevitable in the hardware implementation due to the switching of amplifiers [27–32]. The leakage delays (or forgetting delays) exist in the negative feedback of NNs [33, 34]. These two delays have great impact on the dynamical behaviors of the systems. Simultaneously, time delays can cause oscillation and instability of a system. So it is necessary to adopt some control strategies to stabilize a system. Various types of control methods, such as output-feedback control [35], switching control [36], adaptive control [37] and sampled-data control [38–43] are often considered. In practical applications, the system cannot be in a stable state for a long time, and it is difficult to ensure that the state variables are continuous. Thus, we choose periodic sampling control, which has good flexibility and easy maintenance.

Motivated by the above discussions, the main contributions of this paper can be summarized in the following:

1. We propose a novel model of MMAMNNs, which considers time-varying delays in leakage terms via sampled-data control. Comparing with the previous results, our model combines the characters of both MAMNNs and MNNs, which can simulate the associative memory process of human brains more effectively.
2. The exponential stability and asymptotic stability of equilibrium points for this model are studied. Sufficient criteria guaranteeing the stability of the MMAMNNs with time-varying delays in leakage terms are derived, which based on the Lyapunov functions and some inequality techniques.
3. In practical applications, the system cannot be in a stable state for a long time, and it is difficult to ensure that the state variables are continuous. Thus, we use sampled-data control to ensure the stability of a system in this paper. Compared with continuous control methods, the sample-data control method is more effective and realistic.

The rest of this paper is organized as follows. In the next section, the model of MMAMNNs with time-varying delays in leakage terms via sampled-data control are proposed and some preliminaries are introduced. In section 3, by constructing a suitable Lyapunov function, using the Lyapunov stability theorem and some inequality techniques, some sufficient criteria for ensuring the exponential stability and asymptotic stability of system are obtained. In section 4, numerical examples are given to demonstrate the effectiveness of our results. In section 5, we present our main conclusions.

## 1 Preliminaries

In this section, we consider the following MMAMNNs with time-varying leakage delays:

$$\begin{array}{c} \begin{array}{cc} \frac{dx_{ki}\left(t\right)}{dt} & =I_{ki}\left(t\right)-d_{ki}\left(x_{ki}\left(t\right)\right)x_{ki}\left(t-\gamma_{ki}\left(t\right)\right)+\sum_{\begin{array}{c} p=1,\\ p\neq k \end{array}}^{m}\sum_{j=1}^{n_{p}}a_{pjki}\left(x_{ki}\left(t\right)\right)f_{pj}\left(x_{pj}\left(t\right)\right)\\ & +\sum_{\begin{array}{c} p=1,\\ p\neq k \end{array}}^{m}\sum_{j=1}^{n_{p}}b_{pjki}\left(x_{ki}\left(t\right)\right)g_{pj}\left(x_{pj}\left(t-\tau_{pjki}\left(t\right)\right)\right), \end{array} \end{array}$$ (1)

where *x*_*ki*_(*t*) denotes the voltage of the *ith* neuron in the field *k* at time *t*. *m* is the number of fields in system (1) and *n*_*p*_ corresponds to the number of neurons in the field *p*. *d*_*ki*_(*x*_*ki*_(*t*)), *a*_*pjki*_(*x*_*ki*_(*t*)), *b*_*pjki*_(*x*_*ki*_(*t*)) are connection weights. *f*_*ki*_(*x*) and *g*_*ki*_(*x*) are activation functions. The time delays *γ*_*ki*_(*t*) and *τ*_*pjki*_(*t*) are leakage delays and time-varying delays, respectively. *I*_*ki*_(*t*) represents the sampled-data state feedback inputs of the *ith* neuron in the field *k*.

According to the feature of memristors and the current-voltage characteristic, for convenience, we let

$$\begin{array}{c} \begin{array}{cc} & d_{ki}\left(x_{ki}\left(t\right)\right)=\{\begin{array}{cc} {\overset{´}{d}}_{ki}, & |x_{ki}\left(t\right)|\leq \Gamma_{ki},\\ {\overset{`}{d}}_{ki}, & |x_{ki}\left(t\right)|>\Gamma_{ki}, \end{array}\;\;\;a_{pjki}\left(x_{ki}\left(t\right)\right)=\{\begin{array}{cc} {\overset{´}{a}}_{pjki}, & |x_{ki}\left(t\right)|\leq \Gamma_{ki},\\ {\overset{`}{a}}_{pjki}, & |x_{ki}\left(t\right)|>\Gamma_{ki}, \end{array}\\ & b_{pjki}\left(x_{ki}\left(t\right)\right)=\{\begin{array}{cc} {\overset{´}{b}}_{pjki}, & |x_{ki}\left(t\right)|\leq \Gamma_{ki},\\ {\overset{`}{b}}_{pjki}, & |x_{ki}\left(t\right)|>\Gamma_{ki}, \end{array} \end{array} \end{array}$$ (2)

where the switching jumps Γ_*ki*_ > 0, for *k* = 1, 2, ⋯, *m* and *i* = 1, 2, ⋯, *n*_*k*_. ${\overset{´}{d}}_{ki}>0$, ${\overset{`}{d}}_{ki}>0$, *á*_*pjki*_, *à*_*pjki*_, ${\overset{´}{b}}_{pjki}$, ${\overset{`}{b}}_{pjki}$ are constants.

**Remark 1**. According to the definitions of connection weights, *d*_*ki*_(*x*_*ki*_(*t*)), *a*_*pjki*_(*x*_*ki*_(*t*)) and *b*_*pjki*_(*x*_*ki*_(*t*)) are varying with the state of memristance of system (1). Therefore, we consider the MMAMNNs with time-varying leakage delays as state-dependent switching system. When *d*_*ki*_(*x*_*ki*_(*t*)), *a*_*pjki*_(*x*_*ki*_(*t*)) and *b*_*pjki*_(*x*_*ki*_(*t*)) are constants, system (1) becomes a general MAMNNs.

Because *d*_*ki*_(*x*_*ki*_(*t*)), *a*_*pjki*_(*x*_*ki*_(*t*)) and *b*_*pjki*_(*x*_*ki*_(*t*)) are discontinuities, the solutions considered in this paper are defined in the sense of Filippov. $co[\underline{\xi },\bar{\xi }]$ represent the convex closure on $\left[\underline{\xi },\bar{\xi }\right]$. A column vector is defined as $col\left(x_{ki}\right)=(x_{11},x_{12},\cdots ,x_{1n_{1}},x_{21},\cdots ,x_{mn_{m}}{)}^{T}$. For a continuous function *k*(*t*): *R* → *R*, *D*^+^*k*(*t*) is the upper right Dini derivative of *k*(*t*), and defined as $D^{+}k\left(t\right)={\bar{\text{lim}}}_{h\to 0^{+}}\frac{k(t+h)-k(t)}{h}$. Some notations are defined as follows:

$$\begin{array}{l} {\bar{d}}_{ki}=\text{max}\left\{{\overset{´}{d}}_{ki},{\overset{`}{d}}_{ki}\right\},\;{\underline{d}}_{ki}=\text{min}\left\{{\overset{´}{d}}_{ki},{\overset{`}{d}}_{ki}\right\},\;{\bar{a}}_{pjki}=\text{max}\left\{{\overset{´}{a}}_{pjki},{\overset{`}{a}}_{pjki}\right\},\\ {\underline{a}}_{pjki}=\text{min}\left\{{\overset{´}{a}}_{pjki},{\overset{`}{a}}_{pjki}\right\},\;{\bar{b}}_{pjki}=\text{max}\left\{{\overset{´}{b}}_{pjki},{\overset{`}{b}}_{pjki}\right\},\;{\underline{b}}_{pjki}=\text{min}\left\{{\overset{´}{b}}_{pjki},{\overset{`}{b}}_{pjki}\right\},\\ {\bar{\gamma }}_{ki}=\underset{t\in R}{\text{sup}}\gamma_{ki}\left(t\right),\;{\bar{\tau }}_{pjki}=\underset{t\in R}{\text{sup}}\tau_{pjki}\left(t\right),\;\gamma =\underset{t\in R}{\text{sup}}{\dot{\gamma }}_{ki}\left(t\right),\;\beta =\underset{t\in R}{\text{sup}}{\dot{\tau }}_{pjki}\left(t\right). \end{array}$$

In the Banach space, all sets of continuous functions are expressed as *C*([−*τ*, 0], *R*^*n*^). The initial condition of system (1) are given as follows: $\phi \left(s\right)=(\phi_{11}\left(s\right),\phi_{12}\left(s\right),\cdots ,\phi_{1n_{1}}\left(s\right),\phi_{21}\left(s\right),\cdots ,\phi_{mn_{m}}{\left(s\right))}^{T}\in C\left(\left[-\tau ,0\right],R^{n}\right)$, in which $\tau =\underset{1\leq p\leq m,p\neq k}{\text{max}}\underset{1\leq j\leq n_{p}}{\text{max}}\left\{{\bar{\tau }}_{pjki},{\bar{\gamma }}_{ki}\right\}$.

By applying the set-valued mapping theorem and the differential inclusion theorem, we define the following equations

$$\begin{array}{c} \begin{array}{cc} & co\left(d_{ki}\left(x_{ki}\left(t\right)\right)\right)=\{\begin{array}{cc} {\overset{´}{d}}_{ki}, & |x_{ki}\left(t\right)|<\Gamma_{ki},\\ co\{{\overset{´}{d}}_{ki},{\overset{`}{d}}_{ki}\}, & |x_{ki}\left(t\right)|=\Gamma_{ki},\\ {\overset{`}{d}}_{ki}, & |x_{ki}\left(t\right)|>\Gamma_{ki}, \end{array}\\ & co\left(a_{pjki}\left(x_{ki}\left(t\right)\right)\right)=\{\begin{array}{cc} {\overset{´}{a}}_{pjki}, & |x_{ki}\left(t\right)|<\Gamma_{ki},\\ co\{{\overset{´}{a}}_{pjki},{\overset{`}{a}}_{pjki}\}, & |x_{ki}\left(t\right)|=\Gamma_{ki},\\ {\overset{`}{a}}_{pjki}, & |x_{ki}\left(t\right)|>\Gamma_{ki}, \end{array}\\ & co\left(b_{pjki}\left(x_{ki}\left(t\right)\right)\right)=\{\begin{array}{cc} {\overset{´}{b}}_{pjki}, & |x_{ki}\left(t\right)|<\Gamma_{ki},\\ co\{{\overset{´}{b}}_{pjki},{\overset{`}{b}}_{pjki}\}, & |x_{ki}\left(t\right)|=\Gamma_{ki},\\ {\overset{`}{b}}_{pjki}, & |x_{ki}\left(t\right)|>\Gamma_{ki}. \end{array} \end{array} \end{array}$$ (3)

Obviously, $co\left\{{\overset{´}{d}}_{ki},{\overset{`}{d}}_{ki}\right\}=\left[{\underline{d}}_{ki},{\bar{d}}_{ki}\right]$, $co\left\{{\overset{´}{a}}_{pjki},{\overset{`}{a}}_{pjki}\right\}=[{\underline{a}}_{pjki},{\bar{a}}_{pjki}]$ and $co\left\{{\overset{´}{b}}_{pjki},{\overset{`}{b}}_{pjki}\right\}=\left[{\underline{b}}_{pjki},{\bar{b}}_{pjki}\right]$, for *k*, *p* = 1, 2, ⋯, *m*, *p* ≠ *k*, *i* = 1, 2, ⋯, *n*_*k*_, *j* = 1, 2, ⋯, *n*_*p*_. According to the above definitions, system (1) can be written as follows

$$\begin{array}{c} \begin{array}{cc} \frac{dx_{ki}\left(t\right)}{dt} & \in I_{ki}\left(t\right)-co\left(d_{ki}\left(x_{ki}\left(t\right)\right)\right)x_{ki}\left(t-\gamma_{ki}\left(t\right)\right)+\sum_{\begin{array}{c} p=1,\\ p\neq k \end{array}}^{m}\sum_{j=1}^{n_{p}}co\left(a_{pjki}\left(x_{ki}\left(t\right)\right)\right)f_{pj}\left(x_{pj}\left(t\right)\right)\\ & +\sum_{\begin{array}{c} p=1,\\ p\neq k \end{array}}^{m}\sum_{j=1}^{n_{p}}co\left(b_{pjki}\left(x_{ki}\left(t\right)\right)\right)g_{pj}\left(x_{pj}\left(t-\tau_{pjki}\left(t\right)\right)\right), \end{array} \end{array}$$ (4)

or equivalently, for *k* = 1, 2, ⋯, *m*, *p* ≠ *k*, *i* = 1, 2, ⋯, *n*_*k*_, there exist ${\hat{d}}_{ki}\left(x_{ki}\left(t\right)\right)\in co\left(d_{ki}\left(x_{ki}\left(t\right)\right)\right)$, ${\hat{a}}_{pjki}\left(x_{ki}\left(t\right)\right)\in co\left(a_{pjki}\left(x_{ki}\left(t\right)\right)\right)$, ${\hat{b}}_{pjki}\left(x_{ki}\left(t\right)\right)\in co\left(b_{pjki}\left(x_{ki}\left(t\right)\right)\right)$, such that

$$\begin{array}{c} \begin{array}{cc} \frac{dx_{ki}\left(t\right)}{dt} & =I_{ki}\left(t\right)-{\hat{d}}_{ki}\left(x_{ki}\left(t\right)\right)x_{ki}\left(t-\gamma_{ki}\left(t\right)\right)+\sum_{\begin{array}{c} p=1,\\ p\neq k \end{array}}^{m}\sum_{j=1}^{n_{p}}{\hat{a}}_{pjki}\left(x_{ki}\left(t\right)\right)f_{pj}\left(x_{pj}\left(t\right)\right)\\ & +\sum_{\begin{array}{c} p=1,\\ p\neq k \end{array}}^{m}\sum_{j=1}^{n_{p}}{\hat{b}}_{pjki}\left(x_{ki}\left(t\right)\right)g_{pj}\left(x_{pj}\left(t-\tau_{pjki}\left(t\right)\right)\right). \end{array} \end{array}$$ (5)

**Remark 2**. In [44], the effect of leakage delay on stability was discussed. It was shown that larger leakage delay can lead to instability of a system. In order to reduce the effect of leakage delay, we will use the sampled-data control method to ensure the stability of the system.

In this paper, we consider the following sampled-data controller:

$$\begin{array}{c} I_{ki}\left(t\right)=L_{ki}x_{ki}\left(t_{l}\right), \end{array}$$ (6)

where *L*_*ki*_ denotes the sampled-data feedback control gain matrix, *x*_*ki*_(*t*_*l*_) are discrete measurement of *x*_*ki*_(*t*) at the sampling instant *t*_*l*_. Besides, there exists a constant Δ(Δ > 0) such that *t*_*l*+1_ − *t*_*l*_ ≤ Δ, ∀*l* ∈ *N*, i.e. Δ is the maximum sampling interval. The initial condition becomes *ϕ*(*s*) ∈ *C*([−*τ*, 0], *R*^*n*^), in which $\tau =\underset{1\leq p\leq m,p\neq k}{\text{max}}\underset{1\leq j\leq n_{p}}{\text{max}}\left\{{\bar{\tau }}_{pjki},{\bar{\gamma }}_{ki},\Delta \right\}$.

**Remark 3**. Due to the existence of the discrete term *I*_*ki*_(*t*) = *L*_*ki*_*x*_*ki*_(*t*_*l*_), it is difficult to analyze the stability of system (4). The input delay method was proposed in [45]. By applying it, system (4) will be changed into a continuous system.

The input delay method is applied, we define

$$\begin{array}{c} t_{l}=t-\left(t-t_{l}\right)≔t-\Delta \left(t\right), \end{array}$$ (7)

where 0 ≤ Δ(*t*) < Δ and the sampled-data controller can be written as

$$\begin{array}{c} I_{ki}\left(t\right)=L_{ki}x_{ki}\left(t-\Delta \left(t\right)\right). \end{array}$$ (8)

Then we get

$$\begin{array}{c} \begin{array}{cc} \frac{dx_{ki}\left(t\right)}{dt} & \in L_{ki}x_{ki}\left(t-\Delta \left(t\right)\right)-co\left(d_{ki}\left(x_{ki}\left(t\right)\right)\right)x_{ki}\left(t-\gamma_{ki}\left(t\right)\right)\\ & +\sum_{\begin{array}{c} p=1,\\ p\neq k \end{array}}^{m}\sum_{j=1}^{n_{p}}co\left(a_{pjki}\left(x_{ki}\left(t\right)\right)\right)f_{pj}\left(x_{pj}\left(t\right)\right)\\ & +\sum_{\begin{array}{c} p=1,\\ p\neq k \end{array}}^{m}\sum_{j=1}^{n_{p}}co\left(b_{pjki}\left(x_{ki}\left(t\right)\right)\right)g_{pj}\left(x_{pj}\left(t-\tau_{pjki}\left(t\right)\right)\right). \end{array} \end{array}$$ (9)

Some preliminaries are introduced as follows.

**Assumption 1** For *k* = 1, 2, ⋯, *m*, *i* = 1, 2, ⋯, *n*_*k*_, ∀*s*_1_, *s*_2_ ∈ *R* and *s*_1_ ≠ *s*_2_, the activation functions *f*_*ki*_(⋅) and *g*_*ki*_(⋅) are odd and satisfy a continuous Lipschitz condition, such that

$$\begin{array}{c} \begin{array}{cc} & 0\leq \frac{f_{ki}\left(s_{1}\right)-f_{ki}\left(s_{2}\right)}{s_{1}-s_{2}}\leq \sigma_{ki},\\ & 0\leq \frac{g_{ki}\left(s_{1}\right)-g_{ki}\left(s_{2}\right)}{s_{1}-s_{2}}\leq \rho_{ki}, \end{array} \end{array}$$ (10)

where *σ*_*ki*_ and *ρ*_*ki*_ are nonnegative constants.

**Definition 1** For *k* = 1, ⋯, *m*, *p* ≠ *k*, *i* = 1, ⋯, *n*_*k*_, a constant vector $x^{*}={\left(x_{11}^{*},\cdots ,x_{1n_{1}}^{*},x_{21}^{*},\cdots ,x_{mn_{m}}^{*}\right)}^{T}$ satisfies the following equation

$$\begin{array}{c} \begin{array}{cc} 0\in & -co\left(d_{ki}\left(x_{ki}^{*}\right)\right)x_{ki}^{*}+\sum_{\begin{array}{c} p=1,\\ p\neq k \end{array}}^{m}\sum_{j=1}^{n_{p}}co\left(a_{pjki}\left(x_{ki}^{*}\right)\right)f_{pj}\left(x_{pj}^{*}\right)\\ & +\sum_{\begin{array}{c} p=1,\\ p\neq k \end{array}}^{m}\sum_{j=1}^{n_{p}}co\left(b_{pjki}\left(x_{ki}^{*}\right)\right)g_{pj}\left(x_{pj}^{*}\right)+I_{ki}, \end{array} \end{array}$$ (11)

or equivalently, for *k* = 1, ⋯, *m*, *p* ≠ *k*, *i* = 1, ⋯, *n*_*k*_, there exist ${\hat{d}}_{ki}\left(x_{ki}\left(t\right)\right)\in co\left(d_{ki}\left(x_{ki}\left(t\right)\right)\right)$, ${\hat{a}}_{pjki}\left(x_{ki}\left(t\right)\right)\in co\left(a_{pjki}\left(x_{ki}\left(t\right)\right)\right)$, ${\hat{b}}_{pjki}\left(x_{ki}\left(t\right)\right)\in co\left(b_{pjki}\left(x_{ki}\left(t\right)\right)\right)$, such that

$$\begin{array}{c} \begin{array}{cc} 0= & -{\hat{d}}_{ki}\left(x_{ki}^{*}\right)x_{ki}^{*}+\sum_{\begin{array}{c} p=1,\\ p\neq k \end{array}}^{m}\sum_{j=1}^{n_{p}}{\hat{a}}_{pjki}\left(x_{ki}^{*}\right)f_{pj}\left(x_{pj}^{*}\right)\\ & +\sum_{\begin{array}{c} p=1,\\ p\neq k \end{array}}^{m}\sum_{j=1}^{n_{p}}{\hat{b}}_{pjki}\left(x_{ki}^{*}\right)g_{pj}\left(x_{pj}^{*}\right)+I_{ki}, \end{array} \end{array}$$ (12)

then, the constant vector $x^{*}={\left(x_{11}^{*},x_{12}^{*},\cdots ,x_{1n_{1}}^{*},x_{21}^{*},\cdots ,x_{mn_{m}}^{*}\right)}^{T}$ is an equilibrium point of MMAMNNs with time-varying leakage delays.

**Definition 2** Let the constant vector x* be an equilibrium point of system (1), $x\left(t\right)={\left(x_{11}\left(t\right),\cdots ,x_{1n_{1}}\left(t\right),x_{21}\left(t\right),\cdots ,x_{mn_{m}}\left(t\right)\right)}^{T}$ be an arbitrary solution with the initial condition *ϕ*(*s*) of system (1), if there exist positive constants *β* and *μ* such that $|x\left(t\right)-x^{*}|\leq \mu \text{exp}(-\beta t)\underset{-\tau \leq s\leq 0}{\text{sup}}\left|\phi \left(s\right)-x^{*}\right|,$ then, the equilibrium point x* of system (1) is globally exponential stable.

**Lemma 1** Let Assumption 1 be valid. Then there is at least one local solution *x*(*t*) of system (1) with the initial condition *ϕ*(*s*), *s* ∈ [−*τ*, 0], which is bounded in [46]. Furthermore, the local solution *x*(*t*) of system (1) can be extended to the interval [0, + ∞) in the sense of Filippov.

## Results

In this section, the stability of one equilibrium point will be studied. By constructing a suitable Lyapunov function, some sufficient criteria for exponential stability and asymptotic stability are obtained.

**Theorem 1**. Under Assumption 1, let ${\tilde{d}}_{ki}{\bar{\gamma }}_{ki}<1$, and there exist positive constants $\eta_{11},\eta_{12},\cdots ,\eta_{mn_{m}}$, for *t* > 0, such that the system (9) is globally exponentially stable if

$$\begin{array}{c} \begin{array}{cc} & -\left[{\tilde{d}}_{ki}\left(1-2{\tilde{d}}_{ki}{\bar{\gamma }}_{ki}\right)-{\tilde{d}}_{ki}\gamma -L_{ki}\right]\eta_{ki}/\left(1-{\tilde{d}}_{ki}{\bar{\gamma }}_{ki}\right)+[\sum_{\begin{array}{c} p=1,\\ p\neq k \end{array}}^{m}\sum_{j=1}^{n_{p}}\left|{\tilde{a}}_{pjki}\right|\sigma_{pj}\\ & +\sum_{\begin{array}{c} p=1,\\ p\neq k \end{array}}^{m}\sum_{j=1}^{n_{p}}\left|{\tilde{b}}_{pjki}\right|\rho_{pj}]\eta_{pj}/\left(1-{\tilde{d}}_{pj}{\bar{\gamma }}_{pj}\right)<0, \end{array} \end{array}$$ (13)

where ${\tilde{d}}_{ki}={\overset{´}{d}}_{ki}$ or ${\overset{`}{d}}_{ki}$, ${\tilde{a}}_{pjki}={\overset{´}{a}}_{pjki}$ or *à*_*pjki*_, ${\tilde{b}}_{pjki}={\overset{´}{b}}_{pjki}$ or ${\overset{`}{b}}_{pjki}$. That is, there exists a positive constant λ, which makes |*x*_*ki*_(*t*)| = *O*(*e*^−λ*t*^).

**Proof**. Due to the characteristics of the memristor, the theorem will be proved in three cases.

① |*x*_*ki*_(*t*)| < Γ_*ki*_.

According to the set-valued mapping theorem and the differential inclusion theorem, system (9) can be rewritten as

$$\begin{array}{c} \begin{array}{cc} \frac{dx_{ki}\left(t\right)}{dt} & =L_{ki}x_{ki}\left(t-\Delta \left(t\right)\right)-{\overset{´}{d}}_{ki}x_{ki}\left(t-\gamma_{ki}\left(t\right)\right)+\sum_{\begin{array}{c} p=1,\\ p\neq k \end{array}}^{m}\sum_{j=1}^{n_{p}}{\overset{´}{a}}_{pjki}f_{pj}\left(x_{pj}\left(t\right)\right)\\ & +\sum_{\begin{array}{c} p=1,\\ p\neq k \end{array}}^{m}\sum_{j=1}^{n_{p}}{\overset{´}{b}}_{pjki}g_{pj}\left(x_{pj}\left(t-\tau_{pjki}\left(t\right)\right)\right). \end{array} \end{array}$$ (14)

Then for *ω* > 0, we define a continuous function as follows

$$\begin{array}{c} \begin{array}{cc} \Phi_{ki}\left(\omega \right) & =-[\left({\overset{´}{d}}_{ki}-\omega \right)\left(1-2{\overset{´}{d}}_{ki}{\bar{\gamma }}_{ki}\right)-{\overset{´}{d}}_{ki}\left(e^{\omega {\bar{\gamma }}_{ki}}-\left(1-\gamma \right)\right)-L_{ki}e^{\omega \Delta }]\eta_{ki}/\left(1-{\overset{´}{d}}_{ki}{\bar{\gamma }}_{ki}\right)\\ & +[\sum_{\begin{array}{c} p=1,\\ p\neq k \end{array}}^{m}\sum_{j=1}^{n_{p}}|{\overset{´}{a}}_{pjki}|\sigma_{pj}+e^{\omega {\bar{\tau }}_{pjki}}\sum_{\begin{array}{c} p=1,\\ p\neq k \end{array}}^{m}\sum_{j=1}^{n_{p}}\left|{\overset{´}{b}}_{pjki}\right|\rho_{pj}]\eta_{pj}/\left(1-{\overset{´}{d}}_{pj}{\bar{\gamma }}_{pj}\right)<0. \end{array} \end{array}$$ (15)

According to the condition of Theorem 1, we have

$$\begin{array}{c} \begin{array}{cc} \Phi_{ki}\left(0\right) & =-\left[{\overset{´}{d}}_{ki}\left(1-2{\overset{´}{d}}_{ki}{\bar{\gamma }}_{ki}\right)-{\overset{´}{d}}_{ki}\gamma -L_{ki}\right]\eta_{ki}/\left(1-{\overset{´}{d}}_{ki}{\bar{\gamma }}_{ki}\right)+[\sum_{\begin{array}{c} p=1,\\ p\neq k \end{array}}^{m}\sum_{j=1}^{n_{p}}\left|{\overset{´}{a}}_{pjki}\right|\sigma_{pj}\\ & +\sum_{\begin{array}{c} p=1,\\ p\neq k \end{array}}^{m}\sum_{j=1}^{n_{p}}\left|{\overset{´}{b}}_{pjki}\right|\rho_{pj}]\eta_{pj}/\left(1-{\overset{´}{d}}_{pj}{\bar{\gamma }}_{pj}\right)<0. \end{array} \end{array}$$ (16)

Because Φ_*ki*_(*ω*) is continuous, there exists a small positive constant λ, fulfilling the following in equality

$$\begin{array}{c} \begin{array}{cc} \Phi_{ki}\left(\lambda \right) & =-[\left({\overset{´}{d}}_{ki}-\lambda \right)\left(1-2{\overset{´}{d}}_{ki}{\bar{\gamma }}_{ki}\right)-{\overset{´}{d}}_{ki}\left(e^{\lambda {\bar{\gamma }}_{ki}}-\left(1-\gamma \right)\right)-L_{ki}e^{\lambda \Delta }]\eta_{ki}/\left(1-{\overset{´}{d}}_{ki}{\bar{\gamma }}_{ki}\right)\\ & +[\sum_{\begin{array}{c} p=1,\\ p\neq k \end{array}}^{m}\sum_{j=1}^{n_{p}}|{\overset{´}{a}}_{pjki}|\sigma_{pj}+e^{\lambda {\bar{\tau }}_{pjki}}\sum_{\begin{array}{c} p=1,\\ p\neq k \end{array}}^{m}\sum_{j=1}^{n_{p}}\left|{\overset{´}{b}}_{pjki}\right|\rho_{pj}]\eta_{pj}/\left(1-{\overset{´}{d}}_{pj}{\bar{\gamma }}_{pj}\right)<0. \end{array} \end{array}$$ (17)

We construct a suitable Lyapunov function as follows

$$\begin{array}{c} V_{ki}\left(t\right)=e^{\lambda t}x_{ki}\left(t\right)-\int_{t-\gamma_{ki}\left(t\right)}^{t}{\overset{´}{d}}_{ki}e^{\lambda s}x_{ki}\left(s\right)ds. \end{array}$$ (18)

Calculating the upper right Dini derivative of (18), we obtain

$$\begin{array}{c} \begin{array}{cc} D^{+}V_{ki}\left(t\right) & =\lambda e^{\lambda t}x_{ki}\left(t\right)+e^{\lambda t}{\dot{x}}_{ki}\left(t\right)-{\overset{´}{d}}_{ki}[e^{\lambda t}x_{ki}\left(t\right)-\left(1-{\dot{\gamma }}_{ki}\left(t\right)\right)e^{\lambda (t-\gamma_{ki}\left(t\right))}x_{ki}\left(t-\gamma_{ki}\left(t\right)\right)]\\ & =\lambda e^{\lambda t}x_{ki}\left(t\right)+e^{\lambda t}\{-{\overset{´}{d}}_{ki}x_{ki}\left(t-\gamma_{ki}\left(t\right)\right)+\sum_{\begin{array}{c} p=1,\\ p\neq k \end{array}}^{m}\sum_{j=1}^{n_{p}}{\overset{´}{a}}_{pjki}f_{pj}\left(x_{pj}\left(t\right)\right)\\ & +\sum_{\begin{array}{c} p=1,\\ p\neq k \end{array}}^{m}\sum_{j=1}^{n_{p}}{\overset{´}{b}}_{pjki}g_{pj}\left(x_{pj}\left(t-\tau_{pjki}\left(t\right)\right)\right)+L_{ki}x_{ki}\left(t-\Delta \left(t\right)\right)\}-{\overset{´}{d}}_{ki}e^{\lambda t}x_{ki}\left(t\right)\\ & +{\overset{´}{d}}_{ki}\left(1-{\dot{\gamma }}_{ki}\left(t\right)\right)e^{\lambda (t-\gamma_{ki}\left(t\right))}x_{ki}\left(t-\gamma_{ki}\left(t\right)\right), \end{array} \end{array}$$ (19)

then we get

$$\begin{array}{c} \begin{array}{cc} D^{+}V_{ki}\left(t\right) & =\lambda e^{\lambda t}x_{ki}\left(t\right)-{\overset{´}{d}}_{ki}e^{\lambda t}x_{ki}\left(t\right)+{\overset{´}{d}}_{ki}\left(1-{\dot{\gamma }}_{ki}\left(t\right)\right)e^{\lambda (t-\gamma_{ki}\left(t\right))}x_{ki}\left(t-\gamma_{ki}\left(t\right)\right)\\ & -{\overset{´}{d}}_{ki}e^{\lambda t}x_{ki}\left(t-\gamma_{ki}\left(t\right)\right)+L_{ki}e^{\lambda t}x_{ki}\left(t-\Delta \left(t\right)\right)+e^{\lambda t}[\sum_{\begin{array}{c} p=1,\\ p\neq k \end{array}}^{m}\sum_{j=1}^{n_{p}}{\overset{´}{a}}_{pjki}f_{pj}\left(x_{pj}\left(t\right)\right)\\ & +\sum_{\begin{array}{c} p=1,\\ p\neq k \end{array}}^{m}\sum_{j=1}^{n_{p}}{\overset{´}{b}}_{pjki}g_{pj}\left(x_{pj}\left(t-\tau_{pjki}\left(t\right)\right)\right)]\\ & =-\left({\overset{´}{d}}_{ki}-\lambda \right)V_{ki}\left(t\right)-\left({\overset{´}{d}}_{ki}-\lambda \right)\int_{t-\gamma_{ki}\left(t\right)}^{t}{\overset{´}{d}}_{ki}e^{\lambda s}x_{ki}\left(s\right)ds-[{\overset{´}{d}}_{ki}-{\overset{´}{d}}_{ki}\left(1-{\dot{\gamma }}_{ki}\left(t\right)\right)\\ & \times e^{-\lambda \gamma_{ki}\left(t\right)}]e^{\lambda t}x_{ki}\left(t-\gamma_{ki}\left(t\right)\right)+L_{ki}e^{\lambda t}x_{ki}\left(t-\Delta \left(t\right)\right)+e^{\lambda t}[\sum_{\begin{array}{c} p=1,\\ p\neq k \end{array}}^{m}\sum_{j=1}^{n_{p}}{\overset{´}{a}}_{pjki}\\ & \times f_{pj}\left(x_{pj}\left(t\right)\right)+\sum_{\begin{array}{c} p=1,\\ p\neq k \end{array}}^{m}\sum_{j=1}^{n_{p}}{\overset{´}{b}}_{pjki}g_{pj}\left(x_{pj}\left(t-\tau_{pjki}\left(t\right)\right)\right)]. \end{array} \end{array}$$ (20)

Let $\Omega =\underset{t\in [-\tau ,0]}{\text{sup}}\left|V_{ki}\left(t\right)\right|,\Omega >0$, there exists a positive constant *ξ* such that |*V*_*ki*_(*t*)| ≤ Ω < *ξη*_*ki*_, *t* ∈ [−*τ*, 0]. Then for *t* > 0, we claim that the above formula also holds. The proof will be given as follows.

If the formula is not valid, then there exists a time *t*_0_ > 0, which makes one of the following cases occurring:

$$\begin{array}{c} \begin{array}{c} \left\{ \begin{array}{c} \left[1\right]V_{ki}\left(t_{0}\right)=\xi \eta_{ki},\frac{dV_{ki}\left(t_{0}\right)}{dt}\geq 0,\left|V_{ki}\left(t\right)\right|<\xi \eta_{ki},t<t_{0},\\ \left[2\right]V_{ki}\left(t_{0}\right)=-\xi \eta_{ki},\frac{dV_{ki}\left(t_{0}\right)}{dt}\leq 0,\left|V_{ki}\left(t\right)\right|<\xi \eta_{ki},t<t_{0}. \end{array}\right. \end{array} \end{array}$$ (21)

For *t* < *t*_0_, we have

$$\begin{array}{c} \begin{array}{cc} e^{\lambda t}\left|x_{ki}\left(t\right)\right| & \leq |e^{\lambda t}x_{ki}\left(t\right)-\int_{t-\gamma_{ki}\left(t\right)}^{t}{\overset{´}{d}}_{ki}e^{\lambda s}x_{ki}\left(s\right)ds|+|\int_{t-\gamma_{ki}\left(t\right)}^{t}{\overset{´}{d}}_{ki}e^{\lambda s}x_{ki}\left(s\right)ds|\\ & \leq \xi \eta_{ki}+{\overset{´}{d}}_{ki}{\bar{\gamma }}_{ki}\underset{s\in [-\tau ,t_{0}]}{\text{sup}}e^{\lambda s}\left|x_{ki}\left(s\right)\right|. \end{array} \end{array}$$ (22)

Hence, we get

$$\begin{array}{c} e^{\lambda t}|x_{ki}\left(t\right)|\leq \underset{s\in [-\tau ,t_{0}]}{\text{sup}}e^{\lambda s}\left|x_{ki}\left(s\right)\right|\leq \frac{\xi \eta_{ki}}{1-{\overset{´}{d}}_{ki}{\bar{\gamma }}_{ki}}. \end{array}$$ (23)

For *t* = *t*_0_, system (20) can be written as follows

$$\begin{array}{c} \begin{array}{cc} D^{+}V_{ki}\left(t_{0}\right) & =-\left({\overset{´}{d}}_{ki}-\lambda \right)V_{ki}\left(t_{0}\right)-\left({\overset{´}{d}}_{ki}-\lambda \right)\int_{t-\gamma_{ki}\left(t_{0}\right)}^{t}{\overset{´}{d}}_{ki}e^{\lambda s}x_{ki}\left(s\right)ds-[{\overset{´}{d}}_{ki}\\ & -{\overset{´}{d}}_{ki}\left(1-{\dot{\gamma }}_{ki}\left(t_{0}\right)\right)e^{-\lambda \gamma_{ki}\left(t_{0}\right)}]e^{\lambda t_{0}}x_{ki}\left(t_{0}-\gamma_{ki}\left(t_{0}\right)\right)+L_{ki}e^{\lambda t_{0}}x_{ki}\left(t_{0}-\Delta \left(t_{0}\right)\right)\\ & +e^{\lambda t_{0}}[\sum_{\begin{array}{c} p=1,\\ p\neq k \end{array}}^{m}\sum_{j=1}^{n_{p}}{\overset{´}{a}}_{pjki}f_{pj}\left(x_{pj}\left(t_{0}\right)\right)+\sum_{\begin{array}{c} p=1,\\ p\neq k \end{array}}^{m}\sum_{j=1}^{n_{p}}{\overset{´}{b}}_{pjki}g_{pj}\left(x_{pj}\left(t_{0}-\tau_{pjki}\left(t_{0}\right)\right)\right)]. \end{array} \end{array}$$ (24)

If case [1] occurs, according to Assumption 1 and system (24), we obtain

$$\begin{array}{c} \begin{array}{cc} D^{+}V_{ki}\left(t_{0}\right) & \leq -\left({\overset{´}{d}}_{ki}-\lambda \right)\xi \eta_{ki}+\left({\overset{´}{d}}_{ki}-\lambda \right){\overset{´}{d}}_{ki}{\bar{\gamma }}_{ki}\frac{\xi \eta_{ki}}{1-{\overset{´}{d}}_{ki}{\bar{\gamma }}_{ki}}+[{\overset{´}{d}}_{ki}-{\overset{´}{d}}_{ki}\left(1-{\dot{\gamma }}_{ki}\left(t_{0}\right)\right)\\ & \times e^{-\lambda \gamma_{ki}\left(t_{0}\right)}]e^{\lambda \gamma_{ki}\left(t_{0}\right)}e^{\lambda (t_{0}-\gamma_{ki}\left(t_{0}\right))}x_{ki}\left(t_{0}-\gamma_{ki}\left(t_{0}\right)\right)+L_{ki}e^{\lambda \Delta (t_{0})}\\ & \times e^{\lambda (t_{0}-\Delta \left(t_{0}\right))}x_{ki}\left(t_{0}-\Delta \left(t_{0}\right)\right)+e^{\lambda t_{0}}\sum_{\begin{array}{c} p=1,\\ p\neq k \end{array}}^{m}\sum_{j=1}^{n_{p}}|{\overset{´}{a}}_{pjki}|\sigma_{pj}\left|x_{pj}\left(t_{0}\right)\right|+e^{\lambda \tau_{pjki}\left(t_{0}\right)}\\ & \times \sum_{\begin{array}{c} p=1,\\ p\neq k \end{array}}^{m}\sum_{j=1}^{n_{p}}|{\overset{´}{b}}_{pjki}|\rho_{pj}e^{\lambda (t_{0}-\tau_{pjki}\left(t_{0}\right))}\left|x_{pj}\left(t_{0}-\tau_{pjki}\left(t_{0}\right)\right)\right|\\ & \leq \{-[\left({\overset{´}{d}}_{ki}-\lambda \right)\left(1-2{\overset{´}{d}}_{ki}{\bar{\gamma }}_{ki}\right)-{\overset{´}{d}}_{ki}\left(e^{\lambda {\bar{\gamma }}_{ki}}-\left(1-\gamma \right)\right)-L_{ki}e^{\lambda \Delta }]\frac{\eta_{ki}}{1-{\overset{´}{d}}_{ki}{\bar{\gamma }}_{ki}}\\ & +[\sum_{\begin{array}{c} p=1,\\ p\neq k \end{array}}^{m}\sum_{j=1}^{n_{p}}|{\overset{´}{a}}_{pjki}|\sigma_{pj}+e^{\lambda {\bar{\tau }}_{pjki}}\sum_{\begin{array}{c} p=1,\\ p\neq k \end{array}}^{m}\sum_{j=1}^{n_{p}}\left|{\overset{´}{b}}_{pjki}\right|\rho_{pj}]\frac{\eta_{pj}}{1-{\overset{´}{d}}_{pj}{\bar{\gamma }}_{pj}}\}\xi \\ & =\Phi_{ki}\left(\lambda \right)\xi <0, \end{array} \end{array}$$ (25)

which is contradictory to [1].

Similarly, if case [2] occurs, according to Assumption 1 and system (24), then we obtain

$$\begin{array}{c} \begin{array}{cc} D^{+}V_{ki}\left(t_{0}\right) & \geq \left({\overset{´}{d}}_{ki}-\lambda \right)\xi \eta_{ki}-\left({\overset{´}{d}}_{ki}-\lambda \right){\overset{´}{d}}_{ki}{\bar{\gamma }}_{ki}\frac{\xi \eta_{ki}}{1-{\overset{´}{d}}_{ki}{\bar{\gamma }}_{ki}}-[{\overset{´}{d}}_{ki}-{\overset{´}{d}}_{ki}\left(1-{\dot{\gamma }}_{ki}\left(t_{0}\right)\right)\\ & \times e^{-\lambda \gamma_{ki}\left(t_{0}\right)}]e^{\lambda \gamma_{ki}\left(t_{0}\right)}e^{\lambda (t_{0}-\gamma_{ki}\left(t_{0}\right))}x_{ki}\left(t_{0}-\gamma_{ki}\left(t_{0}\right)\right)-L_{ki}e^{\lambda \Delta (t_{0})}\\ & \times e^{\lambda (t_{0}-\Delta \left(t_{0}\right))}x_{ki}\left(t_{0}-\Delta \left(t_{0}\right)\right)-e^{\lambda t_{0}}\sum_{\begin{array}{c} p=1,\\ p\neq k \end{array}}^{m}\sum_{j=1}^{n_{p}}|{\overset{´}{a}}_{pjki}|\sigma_{pj}\left|x_{pj}\left(t_{0}\right)\right|-e^{\lambda \tau_{pjki}\left(t_{0}\right)}\\ & \times \sum_{\begin{array}{c} p=1,\\ p\neq k \end{array}}^{m}\sum_{j=1}^{n_{p}}|{\overset{´}{b}}_{pjki}|\rho_{pj}e^{\lambda (t_{0}-\tau_{pjki}\left(t_{0}\right))}\left|x_{pj}\left(t_{0}-\tau_{pjki}\left(t_{0}\right)\right)\right|\\ & \geq \{-[\left({\overset{´}{d}}_{ki}-\lambda \right)\left(1-2{\overset{´}{d}}_{ki}{\bar{\gamma }}_{ki}\right)-{\overset{´}{d}}_{ki}\left(e^{\lambda {\bar{\gamma }}_{ki}}-\left(1-\gamma \right)\right)-L_{ki}e^{\lambda \Delta }]\frac{\eta_{ki}}{1-{\overset{´}{d}}_{ki}{\bar{\gamma }}_{ki}}\\ & +[\sum_{\begin{array}{c} p=1,\\ p\neq k \end{array}}^{m}\sum_{j=1}^{n_{p}}|{\overset{´}{a}}_{pjki}|\sigma_{pj}+e^{\lambda {\bar{\tau }}_{pjki}}\sum_{\begin{array}{c} p=1,\\ p\neq k \end{array}}^{m}\sum_{j=1}^{n_{p}}\left|{\overset{´}{b}}_{pjki}\right|\rho_{pj}]\frac{\eta_{pj}}{1-{\overset{´}{d}}_{pj}{\bar{\gamma }}_{pj}}\}\left(-\xi \right)\\ & =-\Phi_{ki}\left(\lambda \right)\xi >0, \end{array} \end{array}$$ (26)

which is contradictory to [2].

In both cases, we know |*V*_*ki*_(*t*)| ≤ Ω < *ξη*_*ki*_, *t* > 0. Similar to (23), we have $e^{\lambda t}|x_{ki}\left(t\right)|\leq \underset{s\in [-\tau ,t_{0}]}{\text{sup}}e^{\lambda s}\left|x_{ki}\left(s\right)\right|\leq \frac{\xi \eta_{ki}}{1-{\overset{´}{d}}_{ki}{\bar{\gamma }}_{ki}},t>0,$ that is, |*x*_*ki*_(*t*)| = *O*(*e*^−λ*t*^). The Theorem 1 is proved.

② |*x*_*ki*_(*t*)| > Γ_*ki*_.

According to the set-valued mapping theorem and the differential inclusion theorem, system (9) can be rewritten as

$$\begin{array}{c} \begin{array}{cc} \frac{dx_{ki}\left(t\right)}{dt} & =L_{ki}x_{ki}\left(t-\Delta \left(t\right)\right)-{\overset{`}{d}}_{ki}x_{ki}\left(t-\gamma_{ki}\left(t\right)\right)+\sum_{\begin{array}{c} p=1,\\ p\neq k \end{array}}^{m}\sum_{j=1}^{n_{p}}{\overset{`}{a}}_{pjki}f_{pj}\left(x_{pj}\left(t\right)\right)\\ & +\sum_{\begin{array}{c} p=1,\\ p\neq k \end{array}}^{m}\sum_{j=1}^{n_{p}}{\overset{`}{b}}_{pjki}g_{pj}\left(x_{pj}\left(t-\tau_{pjki}\left(t\right)\right)\right). \end{array} \end{array}$$ (27)

The proof of the rest is similar to the first case, so it is omitted here.

③ |*x*_*ki*_(*t*)| = Γ_*ki*_.

According to the definition of a convex closure, it is clear that the system (9) is exponentially stable.

In conclusion, the system (9) is exponentially stable under the condition of Theorem 1.

**Remark 4**. According to Assumption 1, it is obvious that (0, 0, ⋯, 0)^*T*^ is a equilibrium point of the system (9).

**Remark 5**. According to the definition of a convex closure, *d*_*ki*_(*x*_*ki*_(*t*)), *a*_*pjki*_(*x*_*ki*_(*t*)) and *b*_*pjki*_(*x*_*ki*_(*t*)) in system (9) are in a interval. Based on the analysis of the first two cases, we know that system (9) is exponentially stable in this interval.

Obviously, system (9) without sample-date feedback control is shown as follows

$$\begin{array}{c} \begin{array}{cc} \frac{dx_{ki}\left(t\right)}{dt} & \in -co\left(d_{ki}\left(x_{ki}\left(t\right)\right)\right)x_{ki}\left(t-\gamma_{ki}\left(t\right)\right)+\sum_{\begin{array}{c} p=1,\\ p\neq k \end{array}}^{m}\sum_{j=1}^{n_{p}}co\left(a_{pjki}\left(x_{ki}\left(t\right)\right)\right)f_{pj}\left(x_{pj}\left(t\right)\right)\\ & +\sum_{\begin{array}{c} p=1,\\ p\neq k \end{array}}^{m}\sum_{j=1}^{n_{p}}co\left(b_{pjki}\left(x_{ki}\left(t\right)\right)\right)g_{pj}\left(x_{pj}\left(t-\tau_{pjki}\left(t\right)\right)\right). \end{array} \end{array}$$ (28)

**Corollary 1**. We consider time-varying delays in the leakage terms without sample-data feedback control. According to Assumption 1, let ${\tilde{d}}_{ki}{\bar{\gamma }}_{ki}<1$, there exist positive constants $\eta_{11},\eta_{12},\cdots ,\eta_{mn_{m}}$, for *t* > 0, such that the equilibrium point x* of system (28) is exponentially stable if

$$\begin{array}{c} \begin{array}{cc} & -\left[{\tilde{d}}_{ki}\left(1-2{\tilde{d}}_{ki}{\bar{\gamma }}_{ki}\right)-{\tilde{d}}_{ki}\gamma \right]\eta_{ki}/\left(1-{\tilde{d}}_{ki}{\bar{\gamma }}_{ki}\right)+[\sum_{\begin{array}{c} p=1,\\ p\neq k \end{array}}^{m}\sum_{j=1}^{n_{p}}\left|{\tilde{a}}_{pjki}\right|\sigma_{pj}\\ & +\sum_{\begin{array}{c} p=1,\\ p\neq k \end{array}}^{m}\sum_{j=1}^{n_{p}}\left|{\tilde{b}}_{pjki}\right|\rho_{pj}]\eta_{pj}/\left(1-{\tilde{d}}_{pj}{\bar{\gamma }}_{pj}\right)<0, \end{array} \end{array}$$ (29)

where ${\tilde{d}}_{ki}={\overset{´}{d}}_{ki}$ or ${\overset{`}{d}}_{ki}$, ${\tilde{a}}_{pjki}={\overset{´}{a}}_{pjki}$ or *à*_*pjki*_, ${\tilde{b}}_{pjki}={\overset{´}{b}}_{pjki}$ or ${\overset{`}{b}}_{pjki}$. That is, there exists a positive constant λ, which makes |*x*_*ki*_(*t*)| = *O*(*e*^−λ*t*^).

**Proof**. Due to the characteristics of the memristor, the theorem will be proved in three cases. The proof process is similar to Theorem 1, and we will not described here.

**Theorem 2**. Under Assumption 1, if there exists a constant λ satisfies

$$\begin{array}{c} L_{ki}^{2}<2\lambda_{ki}-{\tilde{d}}_{ki}^{2}-\sum_{\begin{array}{c} p=1,\\ p\neq k \end{array}}^{m}\sum_{j=1}^{n_{p}}({\tilde{a}}_{pjki}^{2}\sigma_{pj}^{2}+{\tilde{b}}_{pjki}^{2}\rho_{pj}^{2}+1+\frac{1}{1-\beta })-\frac{1}{1-\gamma }-\frac{1}{1-\Delta }, \end{array}$$ (30)

where ${\tilde{d}}_{ki}={\overset{´}{d}}_{ki}$ or ${\overset{`}{d}}_{ki}$, ${\tilde{a}}_{pjki}={\overset{´}{a}}_{pjki}$ or *à*_*pjki*_, ${\tilde{b}}_{pjki}={\overset{´}{b}}_{pjki}$ or ${\overset{`}{b}}_{pjki}$. Then, the solution of system (4) is globally asymptotically stable under the sampled-data controller *I*_*ki*_(*t*) = *L*_*ki*_ *x*_*ki*_(*t* − Δ(*t*)) − λ_*ki*_ *x*_*ki*_(*t*).

**Proof**. We construct a suitable Lyapunov function as follows

$$\begin{array}{c} \begin{array}{cc} V_{ki}\left(t\right) & =\frac{1}{2}x_{ki}^{2}\left(t\right)+\frac{1}{2(1-\gamma )}\int_{t-\gamma_{ki}\left(t\right)}^{t}x_{ki}^{2}\left(s\right)ds+\frac{1}{2(1-\beta )}\\ & \times \sum_{\begin{array}{c} p=1,\\ p\neq k \end{array}}^{m}\sum_{j=1}^{n_{p}}\int_{t-\tau_{pjki}\left(t\right)}^{t}x_{pj}^{2}\left(s\right)ds+\frac{1}{2(1-\Delta )}\int_{t-\Delta (t)}^{t}x_{ki}^{2}\left(s\right)ds. \end{array} \end{array}$$ (31)

Due to the characteristics of the memristor, the theorem will be proved in three cases.

① |*x*_*ki*_(*t*)| < Γ_*ki*_.

According to the set-valued mapping theorem and the differential inclusion theorem, system (9) can be rewritten as follows

$$\begin{array}{c} \begin{array}{cc} \frac{dx_{ki}\left(t\right)}{dt} & =L_{ki}x_{ki}\left(t-\Delta \left(t\right)\right)-\lambda_{ki}x_{ki}\left(t\right)-{\overset{´}{d}}_{ki}x_{ki}\left(t-\gamma_{ki}\left(t\right)\right)+\sum_{\begin{array}{c} p=1,\\ p\neq k \end{array}}^{m}\sum_{j=1}^{n_{p}}{\overset{´}{a}}_{pjki}f_{pj}\left(x_{pj}\left(t\right)\right)\\ & +\sum_{\begin{array}{c} p=1,\\ p\neq k \end{array}}^{m}\sum_{j=1}^{n_{p}}{\overset{´}{b}}_{pjki}g_{pj}\left(x_{pj}\left(t-\tau_{pjki}\left(t\right)\right)\right). \end{array} \end{array}$$ (32)

Calculating the upper right Dini derivative of (32), we have

$$\begin{array}{c} \begin{array}{cc} D^{+}V_{ki}\left(t\right) & =[\frac{1}{2(1-\gamma )}x_{ki}^{2}\left(t\right)-\frac{1}{2}x_{ki}^{2}\left(t-\gamma_{ki}\left(t\right)\right)]+\sum_{\begin{array}{c} p=1,\\ p\neq k \end{array}}^{m}\sum_{j=1}^{n_{p}}[\frac{1}{2(1-\beta )}x_{pj}^{2}\left(t\right)\\ & -\frac{1}{2}x_{pj}^{2}\left(t-\tau \left(t\right)\right)]+[\frac{1}{2(1-\Delta )}x_{ki}^{2}\left(t\right)-\frac{1}{2}x_{ki}^{2}\left(t-\Delta \left(t\right)\right)]+x_{ki}\left(t\right){\dot{x}}_{ki}\left(t\right)\\ & =[\frac{1}{2(1-\gamma )}x_{ki}^{2}\left(t\right)-\frac{1}{2}x_{ki}^{2}\left(t-\gamma_{ki}\left(t\right)\right)]+\sum_{\begin{array}{c} p=1,\\ p\neq k \end{array}}^{m}\sum_{j=1}^{n_{p}}[\frac{1}{2(1-\beta )}x_{pj}^{2}\left(t\right)\\ & -\frac{1}{2}x_{pj}^{2}\left(t-\tau \left(t\right)\right)]+[\frac{1}{2(1-\Delta )}x_{ki}^{2}\left(t\right)-\frac{1}{2}x_{ki}^{2}\left(t-\Delta \left(t\right)\right)]\\ & +x_{ki}\left(t\right)\{L_{ki}x_{ki}\left(t-\Delta \left(t\right)\right)-\lambda_{ki}x_{ki}\left(t\right)-{\overset{´}{d}}_{ki}x_{ki}\left(t-\gamma_{ki}\left(t\right)\right)\\ & +\sum_{\begin{array}{c} p=1,\\ p\neq k \end{array}}^{m}\sum_{j=1}^{n_{p}}{\overset{´}{a}}_{pjki}f_{pj}\left(x_{pj}\left(t\right)\right)+\sum_{\begin{array}{c} p=1,\\ p\neq k \end{array}}^{m}\sum_{j=1}^{n_{p}}{\overset{´}{b}}_{pjki}g_{pj}\left(x_{pj}\left(t-\tau_{pjki}\left(t\right)\right)\right)\}. \end{array} \end{array}$$ (33)

According to Assumption 1, we yield

$$\begin{array}{c} \begin{array}{cc} D^{+}V_{ki}\left(t\right) & \leq [\frac{1}{2(1-\gamma )}x_{ki}^{2}\left(t\right)-\frac{1}{2}x_{ki}^{2}\left(t-\gamma_{ki}\left(t\right)\right)]+\sum_{\begin{array}{c} p=1,\\ p\neq k \end{array}}^{m}\sum_{j=1}^{n_{p}}[\frac{1}{2(1-\beta )}x_{pj}^{2}\left(t\right)\\ & -\frac{1}{2}x_{pj}^{2}\left(t-\tau \left(t\right)\right)]+[\frac{1}{2(1-\Delta )}x_{ki}^{2}\left(t\right)-\frac{1}{2}x_{ki}^{2}\left(t-\Delta \left(t\right)\right)]\\ & +{\overset{´}{d}}_{ki}|x_{ki}\left(t\right)||x_{ki}\left(t-\gamma_{ki}\left(t\right)\right)\left|+\right|x_{ki}\left(t\right)|\sum_{\begin{array}{c} p=1,\\ p\neq k \end{array}}^{m}\sum_{j=1}^{n_{p}}|{\overset{´}{a}}_{pjki}|\sigma_{pj}|x_{pj}\left(t\right)|+|x_{ki}\left(t\right)|\\ & \times \sum_{\begin{array}{c} p=1,\\ p\neq k \end{array}}^{m}\sum_{j=1}^{n_{p}}|{\overset{´}{b}}_{pjki}|\rho_{pj}|x_{pj}\left(t-\tau \left(t\right)\right)|+|x_{ki}\left(t\right)|L_{ki}\left|x_{ki}\left(t-\Delta \left(t\right)\right)\right|-\lambda_{ki}x_{ki}^{2}\left(t\right). \end{array} \end{array}$$ (34)

By the mean-value inequality, we get

$$\begin{array}{c} d_{ki}|x_{ki}\left(t\right)||x_{ki}\left(t-\gamma_{ki}\left(t\right)\right)|\leq \frac{1}{2}[d_{ki}^{2}x_{ki}^{2}\left(t\right)+x_{ki}^{2}\left(t-\gamma_{ki}\left(t\right)\right)], \end{array}$$ (35)

$$\begin{array}{c} |x_{ki}\left(t\right)|\sum_{\begin{array}{c} p=1,\\ p\neq k \end{array}}^{m}\sum_{j=1}^{n_{p}}|{\overset{´}{a}}_{pjki}|\sigma_{pj}\left|x_{pj}\left(t\right)\right|\leq \sum_{\begin{array}{c} p=1,\\ p\neq k \end{array}}^{m}\sum_{j=1}^{n_{p}}[\frac{1}{2}{\overset{´}{a}}_{pjki}^{2}\sigma_{pj}^{2}x_{ki}^{2}\left(t\right)+\frac{1}{2}x_{pj}^{2}\left(t\right)], \end{array}$$ (36)

$$\begin{array}{c} |x_{ki}\left(t\right)|\sum_{\begin{array}{c} p=1,\\ p\neq k \end{array}}^{m}\sum_{j=1}^{n_{p}}|{\overset{´}{b}}_{pjki}|\rho_{pj}\left|x_{pj}\left(t-\tau \left(t\right)\right)\right|\leq \sum_{\begin{array}{c} p=1,\\ p\neq k \end{array}}^{m}\sum_{j=1}^{n_{p}}[\frac{1}{2}{\overset{´}{b}}_{pjki}^{2}\rho_{pj}^{2}x_{ki}^{2}\left(t\right)+\frac{1}{2}x_{pj}^{2}\left(t-\tau \left(t\right)\right)], \end{array}$$ (37)

$$\begin{array}{c} |x_{ki}\left(t\right)|L_{ki}\left|x_{ki}\left(t-\Delta \left(t\right)\right)\right|\leq \frac{1}{2}L_{ki}^{2}x_{ki}^{2}\left(t\right)+\frac{1}{2}x_{ki}^{2}\left(t-\Delta \left(t\right)\right). \end{array}$$ (38)

According to (34) and the mean-value inequality, we get an inequation as follows

$$\begin{array}{c} \begin{array}{cc} D^{+}V_{ki}\left(t\right) & \leq [\frac{1}{2(1-\gamma )}x_{ki}^{2}\left(t\right)-\frac{1}{2}x_{ki}^{2}\left(t-\gamma_{ki}\left(t\right)\right)]+\sum_{\begin{array}{c} p=1,\\ p\neq k \end{array}}^{m}\sum_{j=1}^{n_{p}}[\frac{1}{2(1-\beta )}x_{pj}^{2}\left(t\right)\\ & -\frac{1}{2}x_{pj}^{2}\left(t-\tau \left(t\right)\right)]+[\frac{1}{2(1-\Delta )}x_{ki}^{2}\left(t\right)-\frac{1}{2}x_{ki}^{2}\left(t-\Delta \left(t\right)\right)]+\frac{1}{2}[{\overset{´}{d}}_{ki}^{2}x_{ki}^{2}\left(t\right)\\ & +x_{ki}^{2}\left(t-\gamma_{ki}\left(t\right)\right)]+\sum_{\begin{array}{c} p=1,\\ p\neq k \end{array}}^{m}\sum_{j=1}^{n_{p}}[\frac{1}{2}{\overset{´}{a}}_{pjki}^{2}\sigma_{pj}^{2}x_{ki}^{2}\left(t\right)+\frac{1}{2}x_{pj}^{2}\left(t\right)]\\ & +\sum_{\begin{array}{c} p=1,\\ p\neq k \end{array}}^{m}\sum_{j=1}^{n_{p}}[\frac{1}{2}{\overset{´}{b}}_{pjki}^{2}\rho_{pj}^{2}x_{ki}^{2}\left(t\right)+\frac{1}{2}x_{pj}^{2}\left(t-\tau \left(t\right)\right)]+\frac{1}{2}L_{ki}^{2}x_{ki}^{2}\left(t\right)\\ & +\frac{1}{2}x_{ki}^{2}\left(t-\Delta \left(t\right)\right)-\lambda_{ki}x_{ki}^{2}\left(t\right). \end{array} \end{array}$$ (39)

Then we have

$$\begin{array}{c} \begin{array}{cc} D^{+}V_{ki}\left(t\right) & \leq \{\frac{1}{2}{\overset{´}{d}}_{ki}^{2}+\frac{1}{2}L_{ki}^{2}+\sum_{\begin{array}{c} p=1,\\ p\neq k \end{array}}^{m}\sum_{j=1}^{n_{p}}[\frac{1}{2}{\overset{´}{a}}_{pjki}^{2}\sigma_{pj}^{2}+\frac{1}{2}{\overset{´}{b}}_{pjki}^{2}\rho_{pj}^{2}+\frac{1}{2}+\frac{1}{2(1-\beta )}]\\ & +\frac{1}{2(1-\gamma )}+\frac{1}{2(1-\Delta )}-\lambda_{ki}\}x_{ki}^{2}\left(t\right). \end{array} \end{array}$$ (40)

According to the condition of Theorem 2, we get *D*^+^*V*_*ki*_(*t*)<0. From the Lyapunov stability throrem, the solution of system (9) is globally asymptotically stable.

② |*x*_*ki*_(*t*)| > Γ_*ki*_.

According to the set-valued mapping theorem and the differential inclusion theorem, system (9) can be rewritten as follows

$$\begin{array}{c} \begin{array}{cc} \frac{dx_{ki}\left(t\right)}{dt} & =L_{ki}x_{ki}\left(t-\Delta \left(t\right)\right)-\lambda_{ki}x_{ki}^{2}\left(t\right)-{\overset{`}{d}}_{ki}x_{ki}\left(t-\gamma_{ki}\left(t\right)\right)+\sum_{\begin{array}{c} p=1,\\ p\neq k \end{array}}^{m}\sum_{j=1}^{n_{p}}{\overset{`}{a}}_{pjki}f_{pj}\left(x_{pj}\left(t\right)\right)\\ & +\sum_{\begin{array}{c} p=1,\\ p\neq k \end{array}}^{m}\sum_{j=1}^{n_{p}}{\overset{`}{b}}_{pjki}g_{pj}\left(x_{pj}\left(t-\tau_{pjki}\left(t\right)\right)\right). \end{array} \end{array}$$ (41)

The proof of the rest is similar to the first case, so it is omitted here.

③ |*x*_*ki*_(*t*)| = Γ_*ki*_.

According to the definition of a convex closure, it is clear that the solution of system (9) is globally asymptotically stable.

In conclusion, the solution of system (9) is globally asymptotically stable under the condition of Theorem 2.

Obviously, system (9) without time-varying delays in leakage terms is shown as follows

$$\begin{array}{c} \begin{array}{cc} \frac{dx_{ki}\left(t\right)}{dt} & \in L_{ki}x_{ki}\left(t-\Delta \left(t\right)\right)-\lambda_{ki}x_{ki}\left(t\right)-co\left(d_{ki}\left(x_{ki}\left(t\right)\right)\right)x_{ki}\left(t\right)\\ & +\sum_{\begin{array}{c} p=1,\\ p\neq k \end{array}}^{m}\sum_{j=1}^{n_{p}}co\left(a_{pjki}\left(x_{ki}\left(t\right)\right)\right)f_{pj}\left(x_{pj}\left(t\right)\right)\\ & +\sum_{\begin{array}{c} p=1,\\ p\neq k \end{array}}^{m}\sum_{j=1}^{n_{p}}co\left(b_{pjki}\left(x_{ki}\left(t\right)\right)\right)g_{pj}\left(x_{pj}\left(t-\tau_{pjki}\left(t\right)\right)\right). \end{array} \end{array}$$ (42)

**Corollary 2**. We consider the sample-data feedback control without time-varying delays in the leakage terms. According to Assumption 1, the solution of the system (42) is globally asymptotically stable under the sampled-data controller *I*_*ki*_(*t*) = *L*_*ki*_*x*_*ki*_(*t* − Δ(*t*)) − λ_*ki*_*x*_*ki*_(*t*) if

$$\begin{array}{c} L_{ki}^{2}<2\lambda_{ki}+2{\tilde{d}}_{ki}-\sum_{\begin{array}{c} p=1,\\ p\neq k \end{array}}^{m}\sum_{j=1}^{n_{p}}({\tilde{a}}_{pjki}^{2}\sigma_{pj}^{2}+{\tilde{b}}_{pjki}^{2}\rho_{pj}^{2}+1+\frac{1}{1-\beta })-\frac{1}{1-\Delta }, \end{array}$$ (43)

where ${\tilde{d}}_{ki}={\overset{´}{d}}_{ki}$ or ${\overset{`}{d}}_{ki}$, ${\tilde{a}}_{pjki}={\overset{´}{a}}_{pjki}$ or *à*_*pjki*_, ${\tilde{b}}_{pjki}={\overset{´}{b}}_{pjki}$ or ${\overset{`}{b}}_{pjki}$.

**Proof**. We construct a suitable Lyapunov function as follows

$$\begin{array}{c} \begin{array}{cc} V_{ki}\left(t\right) & =\frac{1}{2}x_{ki}^{2}\left(t\right)+\frac{1}{2(1-\beta )}\sum_{\begin{array}{c} p=1,\\ p\neq k \end{array}}^{m}\sum_{j=1}^{n_{p}}\int_{t-\tau_{pjki}\left(t\right)}^{t}x_{pj}^{2}\left(s\right)ds+\frac{1}{2(1-\Delta )}\int_{t-\Delta (t)}^{t}x_{ki}^{2}\left(s\right)ds. \end{array} \end{array}$$ (44)

Due to the characteristics of the memristor, the corollary will be proved in three cases.

① |*x*_*ki*_(*t*)| < Γ_*ki*_.

According to the set-valued mapping theorem and the differential inclusion theorem, the system (42) can be rewritten as follows

$$\begin{array}{c} \begin{array}{cc} \frac{dx_{ki}\left(t\right)}{dt} & =L_{ki}x_{ki}\left(t-\Delta \left(t\right)\right)-\lambda_{ki}x_{ki}\left(t\right)-{\overset{´}{d}}_{ki}x_{ki}\left(t\right)+\sum_{\begin{array}{c} p=1,\\ p\neq k \end{array}}^{m}\sum_{j=1}^{n_{p}}{\overset{´}{a}}_{pjki}f_{pj}\left(x_{pj}\left(t\right)\right)\\ & +\sum_{\begin{array}{c} p=1,\\ p\neq k \end{array}}^{m}\sum_{j=1}^{n_{p}}{\overset{´}{b}}_{pjki}g_{pj}\left(x_{pj}\left(t-\tau_{pjki}\left(t\right)\right)\right). \end{array} \end{array}$$ (45)

Under the condition of Corollary 2, the proof method is similar to Theorem 2, and we will not described here.

② |*x*_*ki*_(*t*)| > Γ_*ki*_.

According to the set-valued mapping theorem and the differential inclusion theorem, the system (42) can be rewritten as follows

$$\begin{array}{c} \begin{array}{cc} \frac{dx_{ki}\left(t\right)}{dt} & =L_{ki}x_{ki}\left(t-\Delta \left(t\right)\right)-\lambda_{ki}x_{ki}\left(t\right)-{\overset{`}{d}}_{ki}x_{ki}\left(t\right)+\sum_{\begin{array}{c} p=1,\\ p\neq k \end{array}}^{m}\sum_{j=1}^{n_{p}}{\overset{`}{a}}_{pjki}f_{pj}\left(x_{pj}\left(t\right)\right)\\ & +\sum_{\begin{array}{c} p=1,\\ p\neq k \end{array}}^{m}\sum_{j=1}^{n_{p}}{\overset{`}{b}}_{pjki}g_{pj}\left(x_{pj}\left(t-\tau_{pjki}\left(t\right)\right)\right). \end{array} \end{array}$$ (46)

The proof of the rest is similar to the first case, so it will not repeated here.

③ |*x*_*ki*_(*t*)| = Γ_*ki*_.

According to the definition of a convex closure, it is clear that the solution of system (42) is globally asymptotically stable.

In conclusion, the solution of the system (42) without time-varying delays in leakage terms is globally asymptotically stable under the condition of Corollary 2.

## Numerical simulation

In this section, several numerical examples are given to illustrate the efficiency of our theoretical results.

**Example 1**. Consider the following MMAMNNs with leakage delays via sampled-data feedback control, there are three fields and one neuron in each field

$$\begin{array}{c} \begin{array}{c} \left\{ \begin{array}{cc} \frac{dx_{11}\left(t\right)}{dt} & =-d_{11}\left(x_{11}\left(t\right)\right)x_{11}\left(t-\gamma_{11}\left(t\right)\right)+a_{2111}\left(x_{11}\left(t\right)\right)f_{21}\left(x_{21}\left(t\right)\right)\\ & +a_{3111}\left(x_{11}\left(t\right)\right)f_{31}\left(x_{31}\left(t\right)\right)+b_{2111}\left(x_{11}\left(t\right)\right)g_{21}\left(x_{21}\left(t-\tau_{2111}\left(t\right)\right)\right)\\ & +b_{3111}\left(x_{11}\left(t\right)\right)g_{31}\left(x_{31}\left(t-\tau_{3111}\left(t\right)\right)\right)+L_{11}x_{11}\left(t-\Delta \left(t\right)\right),\\ \frac{dx_{21}\left(t\right)}{dt} & =-d_{21}\left(x_{21}\left(t\right)\right)x_{21}\left(t-\gamma_{21}\left(t\right)\right)+a_{1121}\left(x_{21}\left(t\right)\right)f_{11}\left(x_{11}\left(t\right)\right)\\ & +a_{3121}\left(x_{21}\left(t\right)\right)f_{31}\left(x_{31}\left(t\right)\right)+b_{1121}\left(x_{21}\left(t\right)\right)g_{11}\left(x_{11}\left(t-\tau_{1121}\left(t\right)\right)\right)\\ & +b_{3121}\left(x_{21}\left(t\right)\right)g_{31}\left(x_{31}\left(t-\tau_{3121}\left(t\right)\right)\right)+L_{22}x_{22}\left(t-\Delta \left(t\right)\right),\\ \frac{dx_{31}\left(t\right)}{dt} & =-d_{31}\left(x_{31}\left(t\right)\right)x_{31}\left(t-\gamma_{31}\left(t\right)\right)+a_{1131}\left(x_{31}\left(t\right)\right)f_{11}\left(x_{11}\left(t\right)\right)\\ & +a_{2131}\left(x_{31}\left(t\right)\right)f_{21}\left(x_{21}\left(t\right)\right)+b_{1131}\left(x_{31}\left(t\right)\right)g_{11}\left(x_{11}\left(t-\tau_{1131}\left(t\right)\right)\right)\\ & +b_{2131}\left(x_{31}\left(t\right)\right)g_{21}\left(x_{21}\left(t-\tau_{2131}\left(t\right)\right)\right)+L_{33}x_{33}\left(t-\Delta \left(t\right)\right), \end{array}\right. \end{array} \end{array}$$ (47)

where

$$\begin{array}{c} \begin{array}{cc} & d_{11}\left(x_{11}\left(t\right)\right)=\{\begin{array}{cc} 2.32, & |x_{11}|\leq \Gamma_{11},\\ 2.56, & |x_{11}|>\Gamma_{11}, \end{array}\;\;d_{21}\left(x_{21}\left(t\right)\right)=\{\begin{array}{cc} 1.73, & |x_{11}|\leq \Gamma_{21},\\ 2.21, & |x_{11}|>\Gamma_{21}, \end{array}\\ & d_{31}\left(x_{31}\left(t\right)\right)=\{\begin{array}{cc} 1.98, & |x_{31}|\leq \Gamma_{31},\\ 2.18, & |x_{31}|>\Gamma_{31}, \end{array}\;\;a_{1121}\left(x_{21}\left(t\right)\right)=\{\begin{array}{cc} 0.48, & |x_{21}\left(t\right)|\leq \Gamma_{21},\\ 0.73, & |x_{21}\left(t\right)|>\Gamma_{21}, \end{array}\\ & a_{1131}\left(x_{31}\left(t\right)\right)=\{\begin{array}{cc} 1.05, & |x_{31}\left(t\right)|\leq \Gamma_{31},\\ 1.21, & |x_{31}\left(t\right)|>\Gamma_{31}, \end{array}\;\;a_{2111}\left(x_{11}\left(t\right)\right)=\{\begin{array}{cc} -2.32, & |x_{11}\left(t\right)|\leq \Gamma_{11},\\ -5.45, & |x_{11}\left(t\right)|>\Gamma_{11}, \end{array}\\ & a_{2131}\left(x_{31}\left(t\right)\right)=\{\begin{array}{cc} -3.24, & |x_{31}\left(t\right)|\leq \Gamma_{31},\\ -2.56, & |x_{31}\left(t\right)|>\Gamma_{31}, \end{array}\;a_{3111}\left(x_{11}\left(t\right)\right)=\{\begin{array}{cc} -2.1, & |x_{11}\left(t\right)|\leq \Gamma_{11},\\ -1.56, & |x_{11}\left(t\right)|>\Gamma_{11}, \end{array}\\ & a_{3121}\left(x_{21}\left(t\right)\right)=\{\begin{array}{cc} -0.8, & |x_{21}\left(t\right)|\leq \Gamma_{21},\\ -0.76, & |x_{21}\left(t\right)|>\Gamma_{21}, \end{array}\;b_{1121}\left(x_{21}\left(t\right)\right)=\{\begin{array}{cc} -0.56, & |x_{21}\left(t\right)|\leq \Gamma_{21},\\ -0.98, & |x_{21}\left(t\right)|>\Gamma_{21}, \end{array}\\ & b_{1131}\left(x_{31}\left(t\right)\right)=\{\begin{array}{cc} -1.73, & |x_{31}\left(t\right)|\leq \Gamma_{31},\\ -3.56, & |x_{31}\left(t\right)|>\Gamma_{31}, \end{array}\;b_{2111}\left(x_{11}\left(t\right)\right)=\{\begin{array}{cc} -0.4, & |x_{11}\left(t\right)|\leq \Gamma_{11},\\ -1.2, & |x_{11}\left(t\right)|>\Gamma_{11}, \end{array}\\ & b_{2131}\left(x_{31}\left(t\right)\right)=\{\begin{array}{cc} -4.33, & |x_{31}\left(t\right)|\leq \Gamma_{31},\\ -6.67, & |x_{31}\left(t\right)|>\Gamma_{31}, \end{array}\;b_{3111}\left(x_{11}\left(t\right)\right)=\{\begin{array}{cc} 0.23, & |x_{11}\left(t\right)|\leq \Gamma_{11},\\ 0.32, & |x_{11}\left(t\right)|>\Gamma_{11}, \end{array}\\ & b_{3121}\left(x_{21}\left(t\right)\right)=\{\begin{array}{cc} 0.47, & |x_{21}\left(t\right)|\leq \Gamma_{21},\\ 0.58, & |x_{21}\left(t\right)|>\Gamma_{21}. \end{array} \end{array} \end{array}$$ (48)

**Fig 1:**
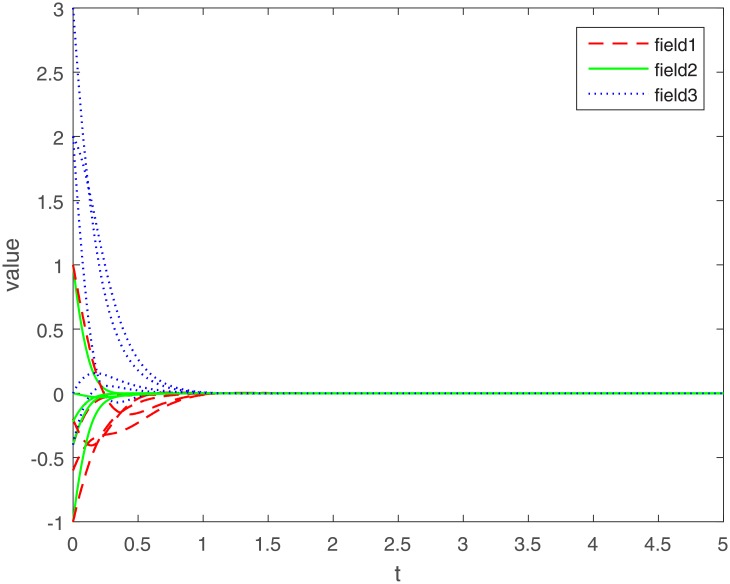
Exponential stability of system (9) with leakage delays via sampled-data feedback control.

**Fig 2:**
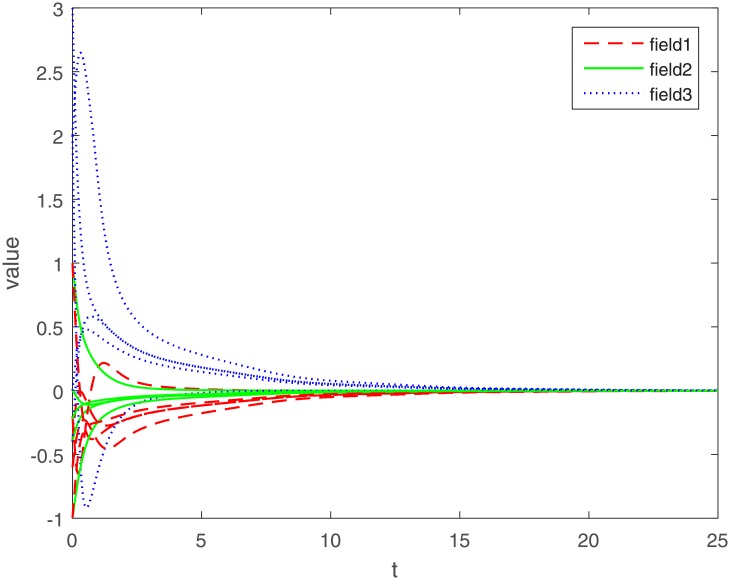
Exponential stability of system (28) without sampled-data feedback control.

**Fig 3:**
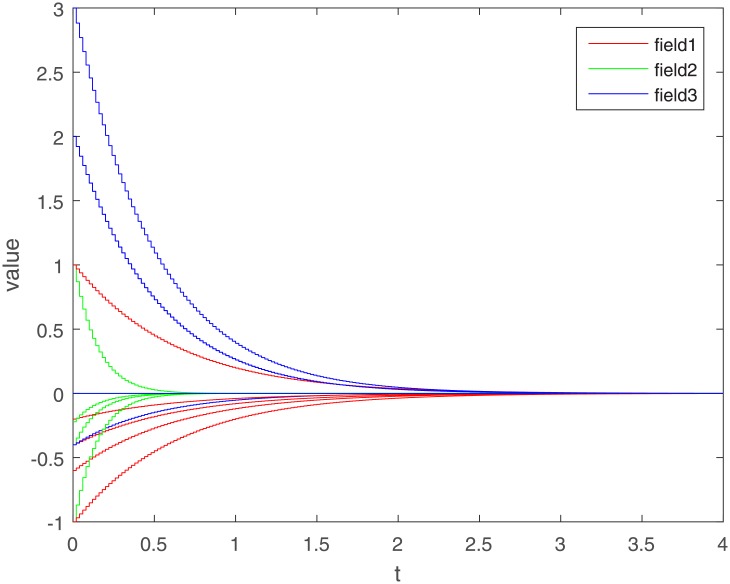
A sampled-data feedback controller for exponential stability of system (9).

**Fig 4:**
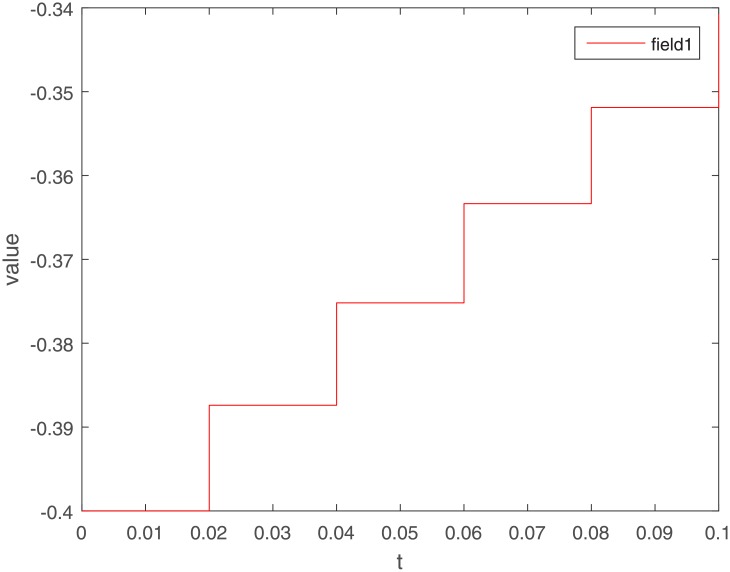
A sampled-data feedback controller for exponential stability of system (9) after local amplification.

Let Γ_11_ = Γ_21_ = Γ_31_ = 2. We set the action functions *f_ki_*(*x*) = *g_ki_*(*x*) = tanh(*x*). The time-varying delays are *γ_ki_*(*t*) = 0.1 + 0.1sin(*t*) and *τ_pjki_* = 0.5cos(*t*) − 0.5. The sampled-data feedback control is set to Δ(*t*) = 0.02*t*. According to Assumption 1, we have *σ*_*ki*_ = *ρ*_*ki*_ = 1. By calculating, we get ${\bar{\gamma }}_{ki}=0.2$, ${\bar{\tau }}_{pjki}=0$, *γ* = 0.1, *β* = 0.5, Δ = 0.02. The initial condition *ϕ*(*s*) ∈ *C*([−0.2, 0], *R*^*n*^). Under the condition of Theorem 1, let *η*_*ki*_ = 2, we get *L*_11_ = −1.6, *L*_21_ = −7, *L*_31_ = −2. The exponential stability of one equilibrium point of the MMAMNNs with time-varying delays in leakage terms via sampled-data feedback control is represented (Fig 1). The exponential stability of one equilibrium point of MMAMNNs with time-varying delays without sampled-data control is showed (Fig 2). A sampled-data feedback controller for exponential stability of system (9) is described (Fig 3). A sampled-data feedback controller for exponential stability of system (9) is described (Fig 4). In the following, five sets of initial values are given

1. *ϕ*_11_ = sin(0.5 * t) − 0.4, *ϕ*_21_ = 0.5 * sin(*t*) − 0.4, *ϕ*_31_ = 0.5 * *t* − 0.4.
2. *ϕ*_11_ = −0.2 + 2 * *t*, *ϕ*_21_ = exp(−0.5 * *t*), *ϕ*_31_ = 2 * cos(*t*).
3. *ϕ*_11_ = exp(0.5 * *t*), *ϕ*_21_ = sin(*t*), *ϕ*_31_ = 3.
4. *ϕ*_11_ = −0.6, *ϕ*_21_ = −1, *ϕ*_31_ = 2.
5. *ϕ*_11_ = −cos(0.5 * *pi* * t), *ϕ*_21_ = −1 + exp(*t* − 0.25), *ϕ*_31_ = 0.2*t.

Under the same parameters, on the one hand, according to Figs 1 and 2, we know that whatever the initial value of each field is, it eventually approximates a straight line. The corresponding value of the line is the equilibrium point value of each field, i.e. no matter what the initial value of each field is, the equilibrium point ultimately converges to zero. In other words, whatever the initial value is, an arbitrary local solution *x*(*t*) is gradually approaching the equilibrium point x* = (0, 0, ⋯, 0)^*T*^.

On the other hand, compared with the MMAMNNs without sample-data control, MMAMNNs with sample-data control converge to the equilibrium point faster. Hence, it is valuable to study the MMAMNNs with time-varying delays in leakage terms via sampled-data feedback control.

**Remark 6**. Since the system cannot be in a stable state for a long time, and it is also a huge consumption to continuously acquire the state of the system, thus, we use sampled-data control method in this paper, which has good flexibility and easy maintenance.

**Fig 5:**
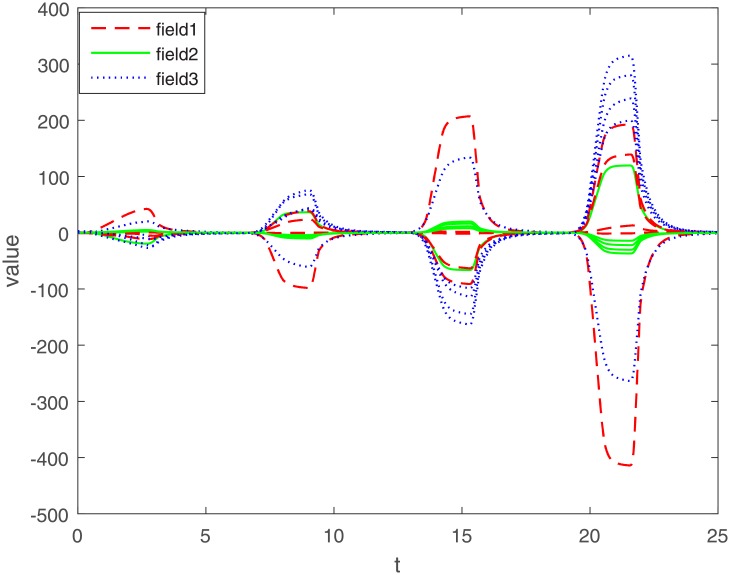
Exponential stability of system (28) without sampled-data feedback control(*γ*_*ki*_(*t*) = 5sin(*t*)).

The varying of MMAMNNs with a larger leakage delay *γ_ki_*(*t*) = 5sin(*t*) and without sample-data control is showed (Fig 5). Compared with Fig 2 with a leakage delay *γ*_*ki*_(*t*) = 0.1 + 0.1sin(*t*), we know that a larger leakage delay can cause fluctuations of system (28) without sampled-data control. Moreover, the system (28) in Fig 5 is unstable and it varies greatly.

**Example 2**. Consider the following MMAMNNs with leakage delays via sampled-data feedback control, there are three fields and one neuron in each field

$$\begin{array}{c} \begin{array}{c} \left\{ \begin{array}{cc} \frac{dx_{11}\left(t\right)}{dt} & =-d_{11}\left(x_{11}\left(t\right)\right)x_{11}\left(t-\gamma_{11}\left(t\right)\right)+a_{2111}\left(x_{11}\left(t\right)\right)f_{21}\left(x_{21}\left(t\right)\right)\\ & +a_{3111}\left(x_{11}\left(t\right)\right)f_{31}\left(x_{31}\left(t\right)\right)+b_{2111}\left(x_{11}\left(t\right)\right)g_{21}\left(x_{21}\left(t-\tau_{2111}\left(t\right)\right)\right)\\ & +b_{3111}\left(x_{11}\left(t\right)\right)g_{31}\left(x_{31}\left(t-\tau_{3111}\left(t\right)\right)\right)+L_{11}x_{11}\left(t-\Delta \left(t\right)\right)-\lambda_{11}x_{11}\left(t\right),\\ \frac{dx_{21}\left(t\right)}{dt} & =-d_{21}\left(x_{21}\left(t\right)\right)x_{21}\left(t-\gamma_{21}\left(t\right)\right)+a_{1121}\left(x_{21}\left(t\right)\right)f_{11}\left(x_{11}\left(t\right)\right)\\ & +a_{3121}\left(x_{21}\left(t\right)\right)f_{31}\left(x_{31}\left(t\right)\right)+b_{1121}\left(x_{21}\left(t\right)\right)g_{11}\left(x_{11}\left(t-\tau_{1121}\left(t\right)\right)\right)\\ & +b_{3121}\left(x_{21}\left(t\right)\right)g_{31}\left(x_{31}\left(t-\tau_{3121}\left(t\right)\right)\right)+L_{22}x_{22}\left(t-\Delta \left(t\right)\right)-\lambda_{21}x_{21}\left(t\right),\\ \frac{dx_{31}\left(t\right)}{dt} & =-d_{31}\left(x_{31}\left(t\right)\right)x_{31}\left(t-\gamma_{31}\left(t\right)\right)+a_{1131}\left(x_{31}\left(t\right)\right)f_{11}\left(x_{11}\left(t\right)\right)\\ & +a_{2131}\left(x_{31}\left(t\right)\right)f_{21}\left(x_{21}\left(t\right)\right)+b_{1131}\left(x_{31}\left(t\right)\right)g_{11}\left(x_{11}\left(t-\tau_{1131}\left(t\right)\right)\right)\\ & +b_{2131}\left(x_{31}\left(t\right)\right)g_{21}\left(x_{21}\left(t-\tau_{2131}\left(t\right)\right)\right)+L_{33}x_{33}\left(t-\Delta \left(t\right)\right)-\lambda_{31}x_{31}\left(t\right), \end{array}\right. \end{array} \end{array}$$ (49)

where

$$\begin{array}{c} \begin{array}{cc} & d_{11}\left(x_{11}\left(t\right)\right)=\{\begin{array}{cc} 2.3, & |x_{11}|\leq \Gamma_{11},\\ 2.8, & |x_{11}|>\Gamma_{11}, \end{array}\;\;d_{21}\left(x_{21}\left(t\right)\right)=\{\begin{array}{cc} 2.1, & |x_{11}|\leq \Gamma_{21},\\ 2.4, & |x_{11}|>\Gamma_{21}, \end{array}\\ & d_{31}\left(x_{31}\left(t\right)\right)=\{\begin{array}{cc} 1.8, & |x_{31}|\leq \Gamma_{31},\\ 2.1, & |x_{31}|>\Gamma_{31}, \end{array}\;\;a_{1121}\left(x_{21}\left(t\right)\right)=\{\begin{array}{cc} -0.72, & |x_{21}\left(t\right)|\leq \Gamma_{21},\\ -0.56, & |x_{21}\left(t\right)|>\Gamma_{21}, \end{array}\\ & a_{1131}\left(x_{31}\left(t\right)\right)=\{\begin{array}{cc} 0.34, & |x_{31}\left(t\right)|\leq \Gamma_{31},\\ 0.78, & |x_{31}\left(t\right)|>\Gamma_{31}, \end{array}\;a_{2111}\left(x_{11}\left(t\right)\right)=\{\begin{array}{cc} 0.25, & |x_{11}\left(t\right)|\leq \Gamma_{11},\\ 0.49, & |x_{11}\left(t\right)|>\Gamma_{11}, \end{array}\\ & a_{2131}\left(x_{31}\left(t\right)\right)=\{\begin{array}{cc} -0.32, & |x_{31}\left(t\right)|\leq \Gamma_{31},\\ -0.16, & |x_{31}\left(t\right)|>\Gamma_{31}, \end{array}\;a_{3111}\left(x_{11}\left(t\right)\right)=\{\begin{array}{cc} 0.96, & |x_{11}\left(t\right)|\leq \Gamma_{11},\\ 1.15, & |x_{11}\left(t\right)|>\Gamma_{11}, \end{array}\\ & a_{3121}\left(x_{21}\left(t\right)\right)=\{\begin{array}{cc} -0.86, & |x_{21}\left(t\right)|\leq \Gamma_{21},\\ -0.54, & |x_{21}\left(t\right)|>\Gamma_{21}, \end{array}\;b_{1121}\left(x_{21}\left(t\right)\right)=\{\begin{array}{cc} 0.78, & |x_{21}\left(t\right)|\leq \Gamma_{21},\\ 0.84, & |x_{21}\left(t\right)|>\Gamma_{21}, \end{array}\\ & b_{1131}\left(x_{31}\left(t\right)\right)=\{\begin{array}{cc} -0.53, & |x_{31}\left(t\right)|\leq \Gamma_{31},\\ -0.17, & |x_{31}\left(t\right)|>\Gamma_{31}, \end{array}\;b_{2111}\left(x_{11}\left(t\right)\right)=\{\begin{array}{cc} 0.58, & |x_{11}\left(t\right)|\leq \Gamma_{11},\\ 0.79, & |x_{11}\left(t\right)|>\Gamma_{11}, \end{array}\\ & b_{2131}\left(x_{31}\left(t\right)\right)=\{\begin{array}{cc} -0.8, & |x_{31}\left(t\right)|\leq \Gamma_{31},\\ -0.65, & |x_{31}\left(t\right)|>\Gamma_{31}, \end{array}\;b_{3111}\left(x_{11}\left(t\right)\right)=\{\begin{array}{cc} 0.68, & |x_{11}\left(t\right)|\leq \Gamma_{11},\\ 1.23, & |x_{11}\left(t\right)|>\Gamma_{11}, \end{array}\\ & b_{3121}\left(x_{21}\left(t\right)\right)=\{\begin{array}{cc} 0.89, & |x_{21}\left(t\right)|\leq \Gamma_{21},\\ 1.01, & |x_{21}\left(t\right)|>\Gamma_{21}. \end{array} \end{array} \end{array}$$ (50)

**Fig 6:**
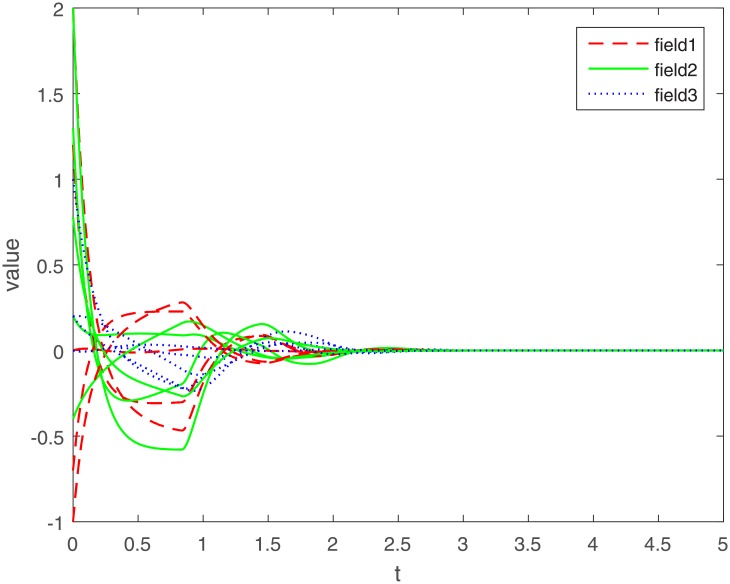
Asymptotic stability of system (9) with leakage delays via sampled-data feedback control.

**Fig 7:**
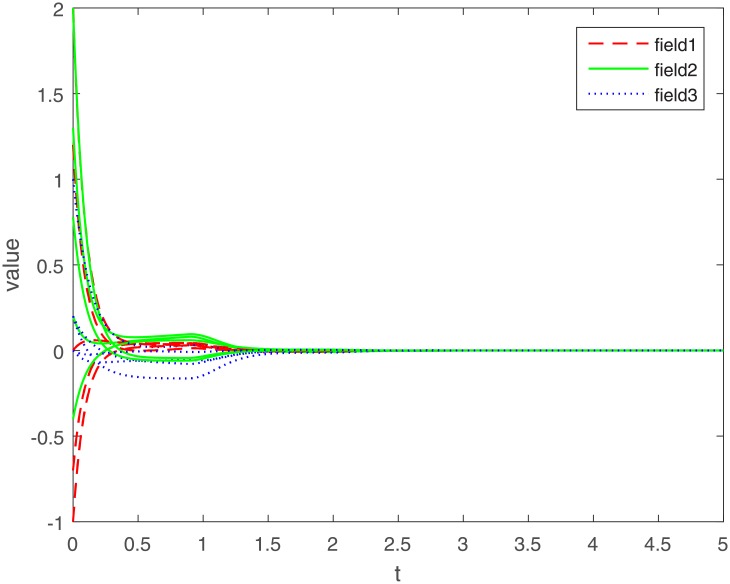
Asymptotic stability of system (42) without leakage terms.

**Fig 8:**
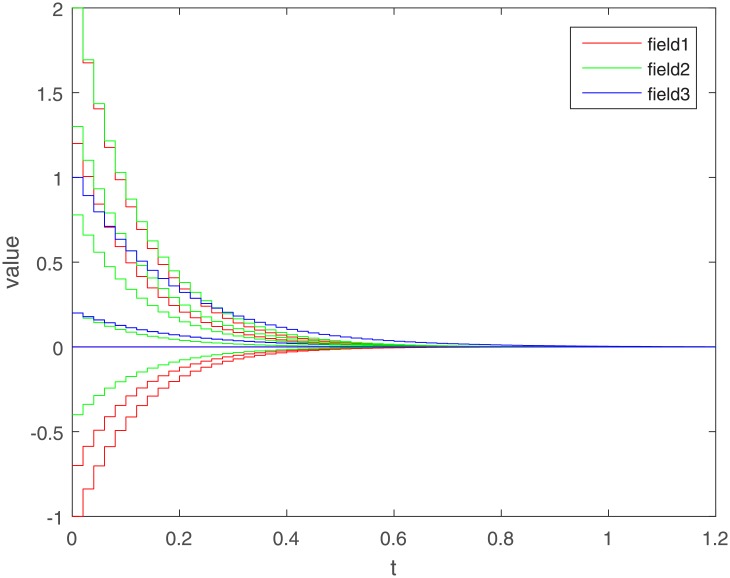
A sampled-data feedback controller for asymptotic stability of system (9).

**Fig 9:**
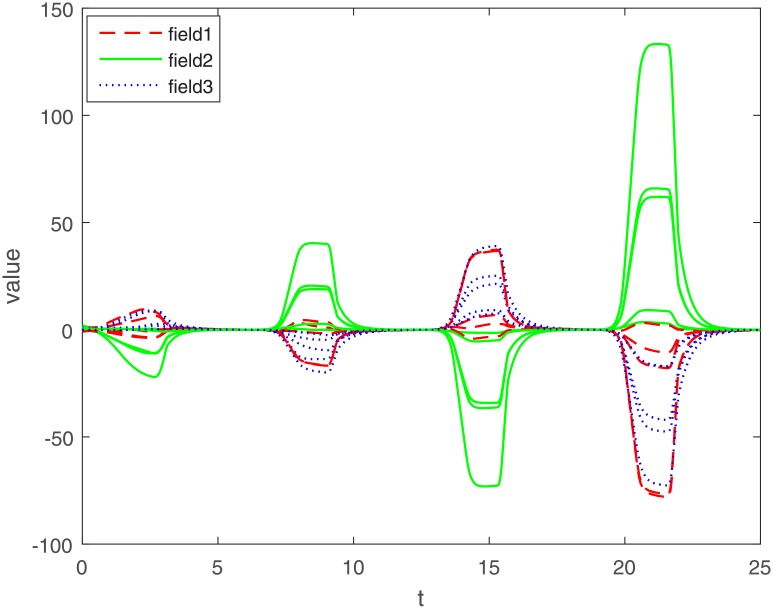
Asymptotic stability of system (9) without sampled-data feedback control(*γ_ki_*(*t*) = 5sin(*t*)).

Let Γ_11_ = Γ_21_ = Γ_31_ = 2. We set the action functions *f_ki_*(*x*) = *g_ki_*(*x*) = tanh(*x*). The time-varying delays are *γ_ki_*(*t*) = 0.5cos(*t*) + 0.5 and *τ*_*pjki*_ = 0.5 + 0.5sin(*t*). The sampled-data feedback control is set to Δ(*t*) = 0.02*t*. According to Assumption 1, we have *σ*_*ki*_ = *ρ*_*ki*_ = 1. By calculating, we get ${\bar{\gamma }}_{ki}=1$, ${\bar{\tau }}_{pjki}=1$, *γ* = 0.5, *β* = 0.5, Δ = 0.02. The initial condition *ϕ*(*s*) ∈ *C*([−1, 0], *R*^*n*^). Under the condition of Theorem 2, let *η*_*ki*_ = 2, we get *L*_11_ = 0.16, *L*_21_ = −0.3, *L*_31_ = 0.32, λ_11_ = 9, λ_21_ = 8, λ_31_ = 6. The asymptotic stability of one equilibrium point of the MMAMNNs with time-varying delays in leakage terms via sampled-data feedback control is displayed (Fig 6). The asymptotic stability of one equilibrium point of MMAMNNs without leakage terms is illustrated (Fig 7). A sampled-data feedback controller for asymptotic stability of system (9) is described (Fig 8). The varying of MMAMNNs with a larger leakage delay *γ_ki_*(*t*) = 5sin(*t*) and without sample-data control is showed (Fig 9). In the following, five sets of initial values are given

1. *ϕ*_11_ = exp(−0.1 * *t*) + 0.2, *ϕ*_21_ = 0.5 * sin(*t*) + 0.2, *ϕ*_31_ = *t* + 0.2.
2. *ϕ*_11_ = 2 * cos(*t*), *ϕ*_21_ = 0.3 + exp(−0.5 * *t*), *ϕ*_31_ = 0.2 + sin(*t*).
3. *ϕ*_11_ = −0.7, *ϕ*_21_ = 2, *ϕ*_31_ = −1 + cos(*t*).
4. *ϕ*_11_ = −0.35*t, *ϕ*_21_ = −0.4 − *t*, *ϕ*_31_ = exp(*t*).
5. *ϕ*_11_ = −cos(0.5 * *pi* * *t*), *ϕ*_21_ = exp(*t* − 0.25), *ϕ*_31_ = 0.2 * tanh(*t*).

Under the same parameters, on the one hand, according to Figs 6 and 7, we know that no matter what the initial value of each field is, it will eventually approach zero. In other words, whatever the initial value is, an arbitrary local solution *x*(*t*) is gradually approaching the equilibrium point x*.

On the other hand, we know that the leakage delays have an effect on the stability of the system. Compared with Figs 6 and 7, it is clear that the curve of MMAMNNs with leakage terms has a significant change. However, the leakage delays are inevitable, so it is significant to study MMAMNNs with leakage terms.

In the simulation experiment, we set the sampling period to 0.02s, and the specific sampling controller action diagram is shown in Fig 4 (after partial enlargement). As can be seen from Figs 3 and 8, the value of the controller remains unchanged during the sampling period until the next sampling period. As time goes on, the system gradually stabilizes and the controller values tend to zero. Compared to continuous control methods, the sampled-data control method reduces energy consumption to a certain extent. At the same time, because the system cannot be in a stable state for a long time, the state of the interval control system is more realistic.

## Conclusion

In this paper, we propose a new model of MMAMNNs with time-varying delays in leakage terms via sampled-data control. Compared with some continuous control methods, the sample-data control method is more effective and realistic. So we turn the sampling system into a continuous time-delay system by using sampled-data control. Then the exponential stability and asymptotic stability of the equilibrium points for this model are analyzed. By constructing a suitable Lyapunov function, using Lyapunov stability theorem and some inequality techniques, some sufficient criteria are obtained to guarantee the stability of the system. Some numerical examples are given to demonstrate the effectiveness of the proposed theories. These results will be further applied in the areas such as associative memory of brain-like systems, intelligent thinking for intelligent robots, mass storage, medical image processing etc.
